# Multi-decadal landscape dynamics and ecological security trajectories driven by 43-year land use changes in Kashgar, an arid border region of Northwest China

**DOI:** 10.1038/s41598-026-51246-y

**Published:** 2026-05-04

**Authors:** Mailikai Aimaiti, Xianyong Meng

**Affiliations:** 1 School of Smart Water Conservancy Engineering, Xinjiang Institute of Technology, Aksu, 843000 China; 2 Univ Gustave Eiffel, ESIEE Paris & COSYS-GRETTIA, 77454 Marne-La-Vall´Ee, France; 3 The Research Center for Climate Change, RCCC, Nanjing, 210029 China

**Keywords:** Land use, Landscape pattern, Landscape ecological security, Spatial autocorrelation analysis, Kashgar region, Ecology, Ecology, Environmental sciences

## Abstract

As a critical ecological-economic nexus along China’s Belt and Road Initiative, the Kashgar region exemplifies tensions between rapid socioeconomic development and ecological fragility in arid Central Asia. Landscape degradation monitoring in arid regions is severely constrained by data scarcity; most studies rely on 15–20 year windows insufficient for detecting decadal-scale threshold behaviors. This study fills a critical research gap by integrating multi-method landscape analysis with a relatively long 43-year land use record (1980–2023). Our integrated analytical framework combines land use dynamic degree, intensity indices, transfer matrices, landscape fragmentation metrics, and spatial autocorrelation analysis, with all results validated through sensitivity analyses (parameterization robustness Spearman rs > 0.96; scale robustness rs > 0.93). We quantified unidirectional anthropogenic landscape reorganization: cultivated and construction land expanded 3,671.78 km^2^ and 973.41 km^2^ respectively, while natural landscapes (forest, grassland, water bodies) collectively declined 2,115.82 km^2^. Construction land displayed the highest transformation intensity (8.41% annual dynamic degree), with peak land use intensity coinciding with China’s Western Development Strategy (2000–2010: Δ*La* = 3.31, *R* = 2.14%). Quantitative landscape fragmentation escalated markedly: patch density increased 68% (0.056 → 0.095/km^2^), shape complexity increased 33% (LSI: 56.097 → 74.590), and spatial connectivity declined 3.25% (CONTAG: 66.870% → 64.698%). Transfer analysis demonstrated that construction land exhibited a persistent unidirectional inflow imbalance, while unused land showed a consistent unidirectional outflow imbalance; other land types underwent bidirectional transitions. Landscape ecological security exhibited distinctive three-phase dynamics (improvement → degradation → recovery) with strong persistent spatial autocorrelation (*Moran’s I*: 0.78–0.81, p < 0.001), generating stable "high-high" clusters in oases and vulnerable "low-low" clusters in desert margins. Quantified attribution analysis (Grey Relational Analysis, Spearman correlations) revealed that GDP growth was the strongest driver of construction land expansion (GRG = 0.87), while population growth was the primary driver of cultivated land expansion (GRG = 0.85). Our preliminary observations suggest a potential monitoring reference zone (Δ*La* ≈ 2.5–3.5 per decade) for early-warning systems; rigorous cross-regional validation is essential before establishing universal thresholds. Persistence of ecological degradation into 2010–2020 despite reduced land use change (*LC*: 1.20% → 0.15%) indicates hysteresis dynamics in arid systems. This 43-year dataset and validated analytical framework provide critical baseline data and evidence-based quantitative thresholds for early-warning systems and territorial spatial planning in ecologically fragile arid zones.

**Supplementary Information:**

The online version contains supplementary material available at 10.1038/s41598-026-51246-y.

## Introduction

Land serves as the most fundamental natural resource and provides the material basis for human survival and development^1^. Land use/cover change (LUCC) represents the most direct manifestation of human–environment interactions^2^, with spatial–temporal variations in land use being closely linked to the intensity of human socio-economic activities^3,4^. LUCC directly influences landscape patterns, while the evolution of landscape patterns conversely serves as the most direct manifestation of LUCC^5,6^. Landscape pattern refers to the spatial distribution and configuration of landscape elements of varying sizes and shapes, reflecting landscape heterogeneity and resulting from the interplay of natural and anthropogenic factors across different spatiotemporal scales^7,8^. Over the long term, the evolution of landscape patterns leads to corresponding changes in spatial structure and function, which are intuitively reflected in ecosystem structure, composition, and ultimately, ecological security^9,10^. Ecological security constitutes a vital component of national and international security, and playing a key role in promoting sustainable economic and social development^11,12^. With the expansion and intensification of human activities, a series of ecological and environmental problems, such as environmental degradation, pollution, resource scarcity, global warming, and flooding, etc., have emerged successively. These challenges have become significant constraints on regional healthy development and pose major obstacles to achieving regional sustainability^13,14^. In the context of accelerated urbanisation and escalating ecological pressures, ecological security has garnered widespread governmental attention worldwide, emerging as a frontier issue in global change research and a persistent focus of academic inquiry^15,16^.

In recent years, research on land use landscape pattern change and ecological security has increasingly emphasized land use landscape pattern changes^17–19^, driving force analysis^20–22^, landscape pattern optimization and dynamic simulation^23–26^, as well as landscape ecological security assessment^10,27,28^. The research objects have included watersheds^10,21,23,29^, wetlands^30–32^, lakes^33–35^, forests^36–38^, mountainous areas^39–41^, urban areas characterised by complex human-land interactions^42–44^, nature reserves^45–47^, and ecologically fragile zones^48,49^. However, three critical research gaps constrain current understanding: (1) Spatial-governance mismatch—while land use policies are implemented at county level, most studies operate at provincial or urban agglomeration scales, creating a disconnect between scientific findings and policy application, particularly in ecologically fragile border regions where governance sensitivity is paramount. The present study addresses this gap at the prefecture scale to establish a comprehensive multi-decadal baseline; county-level disaggregation to directly inform sub-regional governance is identified as a priority direction for future research; (2) Methodological compartmentalization—landscape pattern analysis and ecological security assessment remain methodologically isolated, precluding mechanistic understanding of how pattern changes cascade into security outcomes; (3) Temporal inadequacy—predominant reliance on 15–20 year observational windows fails to capture decadal-scale dynamics essential for detecting threshold behaviors and regime shifts in arid landscape systems. In the Kashgar region, although previous scholars^50,51^ have produced valuable findings regarding landscape pattern evolution, the same limitations persist. To address these research gaps, the present study utilizes land use data from six periods in 1980, 1990, 2000, 2010, 2020, and 2023, combined with GIS technology to examine the dynamic evolution characteristics and changing rules of land use landscape patterns in the Kashgar region over the past 43 years. Specifically, we aim to: (1) characterize multi-decadal trajectories of land use transformation and associated landscape pattern evolution; (2) quantify landscape ecological security dynamics and identify spatial clustering patterns; (3) elucidate coupled human-natural driving mechanisms underlying observed changes; and (4) identify critical thresholds that may inform early-warning systems for ecological degradation. These findings will contribute to evidence-based territorial spatial planning in arid ecologically fragile zones.

## Materials and methods

### Study area

**Fig. 1:**
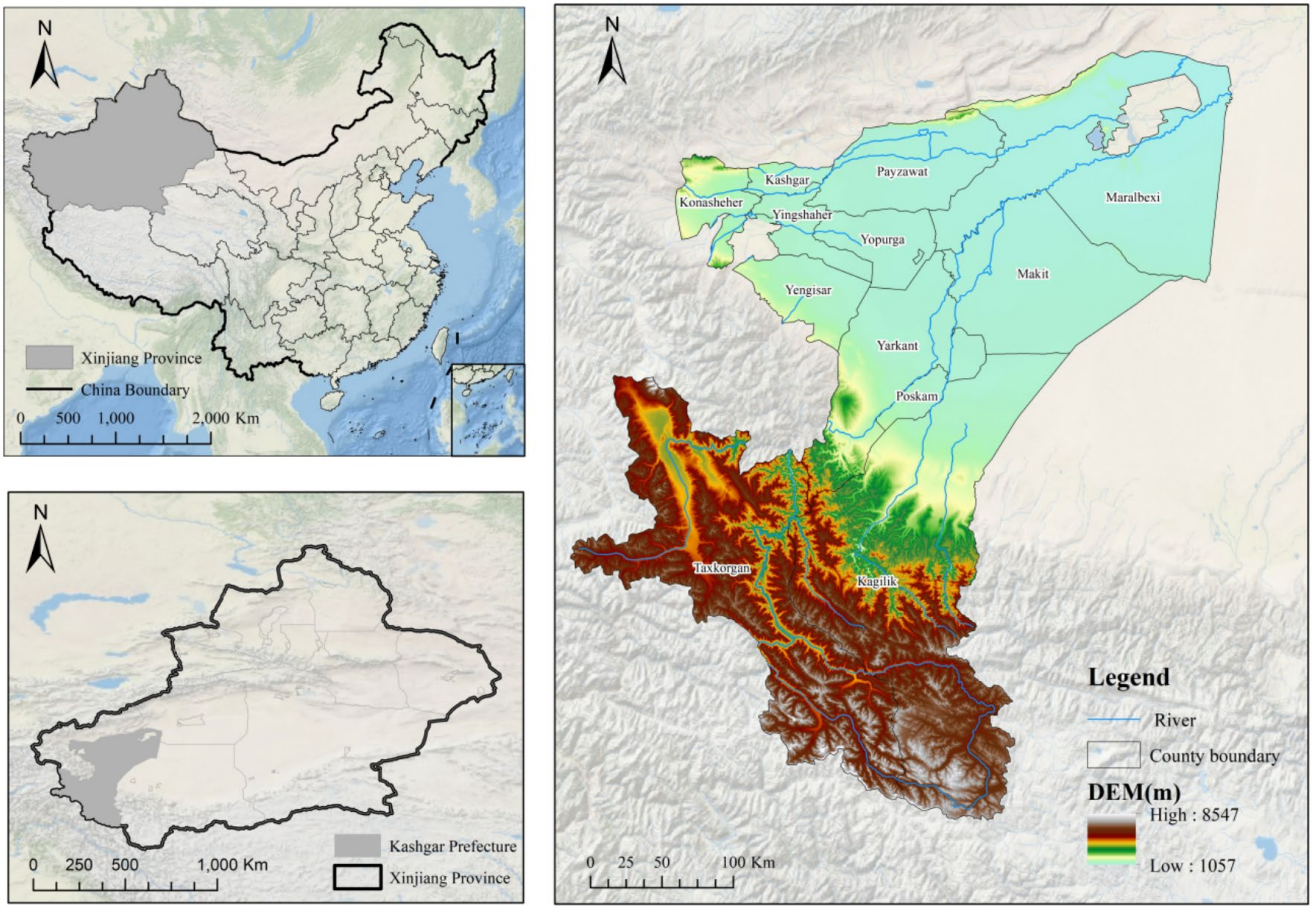
Location map of the study area. The map is generated with ArcGIS software (Version 10.8, https://www.esri.com).

Kashgar region (73°27′–79°57′E, 35°20′–40°18′N) is located in the southwestern part of the Xinjiang Uygur Autonomous Region in northwestern China (Fig. 1). It is bordered by the Taklamakan Desert to the east, the Kizilsu Kirghiz Autonomous Prefecture to the northwest, and Hotan Prefecture to the southeast^52^. As a key economic hub, Kashgar serves as a vital node on the “Silk Road” Economic Belt and a strategic bridge along the China-Pakistan Economic Corridor. The total area of the region is measured at 110,363.69 km2 (according to data on land use area collected for this study). Administratively, the Kashgar region comprises one county-level city, ten counties, and one autonomous county. The climate is characterized by a typical continental arid climate^53^, distinguished by scarce precipitation, long sunshine hours and high evapotranspiration^54^.

By the end of 2023, the population of the study area had reached 4.496 million, accounting for approximately 17.35% of the total population of Xinjiang. The Gross Domestic Product (GDP) was recorded at 150.835 billion CNY. The per capita GDP was 31,520 CNY, representing an increase of 5.9% compared to the previous year.

### Data sources

The data used in this study primarily includes: (1) The LUCC data for 1980, 1990, 2000, 2010, 2020 and 2023 were obtained from the Resource and Environmental Science Data Center (RESDC) of the Chinese Academy of Sciences (http://www.resdc.cn/) with a spatial resolution of 30 m × 30 m, following the classification scheme and validation protocols documented by Xu et al. (2018)^55^. The land use types were classified into six categories following the national standard classification system: cultivated land, forest land, grass land, water bodies, construction land, and unused land. Accuracy assessment of the CNLUCC dataset was validated using field surveys and visual interpretation, with an overall accuracy exceeding 90% as reported by Xu et al. (2018)^55^. (2) Meteorological data including, annual mean temperature, precipitation, and relative humidity were acquired from the National Meteorological Information Centre (http://data.cma.cn). (3) Socioeconomic data were collected from the Kashi Statistical Yearbook (1949–1989), the Xinjiang Statistical Yearbook (1981–2022)^56^. (4) Basic geographic data, including city and county boundaries, roads, and rivers, were sourced from the Resource and Environmental Science Data Cloud Platform (http://www.resdc.cn/)^55^.

### Research methods

#### Land use dynamic degree

The dynamic changes in land use landscape types within the study area were further analyzed using the single and integrated land use dynamic degree.

1. Single land use dynamic degree (*K*) indicates the rate and magnitude of different land use type change in a certain period^57,58^. Its calculation equation is as follows:
  1 $$K = \frac{{\mathop U\nolimits_{b} - \mathop U\nolimits_{a} }}{{\mathop U\nolimits_{a} }} \times \frac{1}{T} \times 100\%$$

where, *U*_*a*_ and *U*_*b*_ respectively denotes the area of a specific land use type at the initial stage and the final stage of the study (km^2^); *T* is the study period.

2) Integrated land use dynamic degree (*LC*) characterizes the overall annual change rate of land use in the study area^59,60^. The calculation equation is as follows:
  2 $$LC = \frac{{\sum\limits_{i = 1}^{n} {\Delta \mathop {LU}\nolimits_{i - j} } }}{{2\sum\limits_{i = 1}^{n} {\mathop {LU}\nolimits_{i} } }} \times \frac{1}{T} \times 100\%$$
  where, *LU*_*i*_ indicates the area (km^2^) of the *i-*th type of land use at the initial stage of the study period; *LU*_*i-j*_ signifies the absolute value of the area converted from land use type *i* to type *j* throughout the duration of the study; *T* refers to study period (a).

#### Land use intensity

Land use intensity index can reflect the degree of human interference on land natural complexes to a certain extent^61,62^. Through the comprehensive index of land use intensity, the amount of land use intensity change, and the change rate index of land use intensity, to analysis the level of integrated land use and its changing trends within the research area^63^.

3 $$La = 100 \times \sum\limits_{i = 1}^{n} {\left( {\mathop A\nolimits_{i} \times \mathop C\nolimits_{i} } \right)} \quad La \in \left[ {{1}00,{4}00} \right]$$

where, *La* denotes the comprehensive index of land use intensity, and its value range is [100,400]; *A*_*i*_ signifies the grading index for the i-th level of land use intensity. The value of *A*_*i*_ was assigned according to the graded indices established in previous studies^64–66^, where different land use types were categorized into four intensity grades: unused land, level 1; forest land, grass land and water body, level 2; cultivated land, level 3; construction land, level 4; *C*_*i*_ represents the area percentage of the *i* th land use type; *n* denotes the number of land use intensity grades.

4 $$\Delta La = \mathop {La}\nolimits_{y} - \mathop {La}\nolimits_{x} = 100 \times \left[ {\sum\limits_{i = 1}^{n} {\left( {\mathop A\nolimits_{i} \times \mathop C\nolimits_{iy} } \right) - \sum\limits_{i = 1}^{n} {\left( {\mathop A\nolimits_{i} \times \mathop C\nolimits_{ix} } \right)} } } \right]$$

5 $$R = \frac{{\sum\limits_{i = 1}^{n} {\left( {\mathop A\nolimits_{i} \times \mathop C\nolimits_{iy} } \right) - \sum\limits_{i = 1}^{n} {\left( {\mathop A\nolimits_{i} \times \mathop C\nolimits_{ix} } \right)} } }}{{\sum\limits_{i = 1}^{n} {\left( {\mathop A\nolimits_{i} \times \mathop C\nolimits_{ix} } \right)} }}$$

where, Δ*La* signifies the amount of land use intensity change, indicating the variation in land use intensity over a specific period; *La*_*x*_* and La*_*y*_ are the comprehensive index of land use intensity at the beginning and end of the research period, respectively; *C*_*iy*_ and *C*_*ix*_ refers to the area percentage of the *i* th level land use type in period *y* and period *x*, respectively; *R* represents the change rate index of land use intensity. If Δ*La* > 0, and *R* > 0, the land use in the region is in a phase of development or sustained utilization.

#### Land use transfer matrix

Land use transfer matrix delineates the dynamic process of interconversion among different land use types in the study area over a specified period^2,67^, and quantitatively describes the transitional directions of each land use type at the beginning of the study period and the sources of each land use type at the end^68^. thus better understanding the spatiotemporal evolution of land use landscape types. The transfer matrix is expressed as follows:

6 $$\mathop S\nolimits_{ij} = \left| {\begin{array}{*{20}c} {\mathop S\nolimits_{11} } & {\mathop S\nolimits_{12} } & {...} & {\mathop S\nolimits_{1n} } \\ {\mathop S\nolimits_{21} } & {\mathop S\nolimits_{22} } & {...} & {\mathop S\nolimits_{2n} } \\ {...} & {...} & {...} & {...} \\ {\mathop S\nolimits_{n1} } & {\mathop S\nolimits_{n2} } & {...} & {\mathop S\nolimits_{nn} } \\ \end{array} } \right|$$

where, *S*_*ij*_ represents the area (km^2^) where land use type *i* at the beginning of the study period is converted to the *j* by the end of the period; *n* represents the number of land use types.

#### Selection of landscape pattern index

Landscape pattern indices quantitatively characterize the structural composition and its spatial configuration of landscape patterns^69^. Based on the actual condition of the Kashgar region and considering correlations among landscape indices^21,70,71^, six landscape pattern indices were selected at both class and landscape levels. To minimize multicollinearity while ensuring comprehensive pattern characterization, we applied Spearman correlation analysis to screen candidate indices (threshold: |r|< 0.7), retaining those capturing distinct pattern dimensions. The selected indices span six orthogonal dimensions: area-dominance (LPI), fragmentation (PD), edge complexity (ED), shape irregularity (LSI), diversity (SHDI), and spatial connectivity (CONTAG), following established protocols for arid landscape assessment.

At the class level, we selected the patch density (PD), largest patch index (LPI), landscape shape index (LSI) and edge density (ED). At the landscape level, were selected: the patch density (PD), largest patch index (LPI), landscape shape index (LSI), edge density (ED), contagion index (CONTAG) and Shannon diversity index (SHDI). The selection of these indices encompasses multiple dimensions of landscape patterns, including landscape fragmentation, complexity, aggregation, and diversity. Through the application of these indices, the landscape pattern characteristics of the Kashgar region can be comprehensively and accurately represented. Detailed computational methods and ecological interpretations of these indices can be found in the relevant literature^6,32,39,43,69,70^.

#### Landscape ecological security evaluation model

##### Landscape disturbance index

Landscape disturbance is a widely used metric for evaluating the extent of ecosystem disruption resulting from both natural and human activities. In this study, the landscape fragmentation index (*C*_*i*_), separation index (*S*_*i*_), and dominance index (*D*_*i*_) were integrated to construct the landscape disturbance index^47,72^. The equation for calculating the landscape disturbance index is as follows:

7 $$\mathop E\nolimits_{i} = \mathop {aC}\nolimits_{i} + \mathop {bS}\nolimits_{i} + \mathop {cD}\nolimits_{i}$$

$$\mathop C\nolimits_{i} = \frac{{N_{i} }}{{A_{i} }}\quad \mathop S\nolimits_{i} = \frac{A}{{2A_{i} }}\sqrt {\frac{{N_{i} }}{A}} \quad \mathop D\nolimits_{i} = \frac{{\mathop Q\nolimits_{i} + \mathop M\nolimits_{i} + \mathop L\nolimits_{i} }}{3}$$

where, *E*_*i*_ is the landscape disturbance index of the *i* th landscape type; *N*_*i*_ and *A*_*i*_ represents the number of patches and the total area of the *i* th landscape type, respectively; *A* denotes the total landscape area of the the study area (km^2^); *Q*_*i*_ indicates the ratio of the units of the *i* th landscape type to the total units; *M*_*i*_ is the ratio of the number of patches of the *i* th landscape type to the total number of patches; *L*_*i*_ is the ratio of the area of the *i* th landscape type to the total study area. *a*, *b*, *c* represent the weights of landscape fragmentation, separation, and dominance, respectively, and *a* + *b* + *c* = 1; The values of *a*, *b* and *c* were established as 0.5, 0.3 and 0.2 respectively, based on the characteristics of the study area and informed by previous research^73^*.*

##### Landscape vulnerability index

Landscape vulnerability index (*F*_*i*_) indicates the sensitivity of various landscape types to external disturbances^72^. Based on previous studies^27,29^, landscape vulnerability was classified into six levels in this research. Although Reference 27 was developed in a mining city context and Reference 29 in a karst watershed, the underlying vulnerability ordering principle — that construction land represents the most stable land use type due to its irreversibility, while unused land and water bodies exhibit the greatest sensitivity to external disturbance — is consistent with arid zone ecological theory and has been applied in comparable dryland landscape studies 73. The relative ordering of vulnerability levels was therefore retained, and weights were derived through linear normalization of the six ordinal levels (Table 1). Construction land was the most stable, and once transferred to construction land, it is less likely to undergo further disturbances. Therefore, the vulnerability level is assigned a value of 1. Forest land and grass land, being natural landscapes with relatively low development pressure, were assigned values of 2 and 3, respectively. Cultivated land, though highly vulnerable to construction land, was assigned a value of 4 due to protection under strict farmland policies. Water body and unused land, being the most vulnerable and sensitive to human disturbance, hence, assigned values of 5 and 6, respectively. The corresponding weights were obtained by normalizing these vulnerability levels (Table 1).

**Table 1:** The weight and vulnerability levels.

Assignment level	land use landscape type					
	Construction land	Forest land	Grass land	Cultivated land	Water body	Unused land
vulnerability level	1	2	3	4	5	6
weight	0.0476	0.0952	0.1429	0.1905	0.2381	0.2857

##### Landscape ecological security index

Considering the patch area characteristics of the study area, a 1 km^2^ × 1 km^2^ grid was established for the Kashgar region using ArcGIS 10.8. This unit size was selected based on three considerations: (1) it substantially exceeds the minimum mapping unit of the 30 m resolution LUCC source data, thereby minimizing sub-pixel classification noise while retaining sufficient spatial detail; (2) it is consistent with the evaluation unit applied in the most directly comparable arid landscape ecological security study in the region (Reference 27), facilitating methodological comparability; and (3) it corresponds to the spatial scale at which landscape pattern metrics (particularly PD and CONTAG) exhibit stable and ecologically meaningful sensitivity in dryland systems. To verify scale robustness, *ES*_*k*_ was additionally computed using 4 km^2^ and 9 km^2^ grid units. The spatial ranking of ecological security levels and Moran’s I values under these alternative units were highly consistent with the 1 km^2^ results (Spearman rs > 0.93, p < 0.001 in all periods; see Table S1 in Supporting Information for detailed comparisons across all grid sizes and weight schemes), confirming that the 1 km^2^ unit produces representative and scale-robust conclusions. Building upon the landscape disturbance and vulnerability index^27,74^, the landscape ecological security index for each grid was calculated through an area-weighted summation approach.

8 $$\mathop {ES}\nolimits_{k} = \sum\limits_{i = 1}^{m} {\frac{{\mathop A\nolimits_{ki} }}{{\mathop A\nolimits_{k} }}} \left( {1 - 10 \times \mathop E\nolimits_{i} \times \mathop F\nolimits_{i} } \right)$$

where, *A*_*ki*_ indicates the area of the *i* th landscape type within the *k* th evaluation unit; *A*_*k*_ indicates the total area of the *k* th evaluation unit; and *m* denotes the total number of landscape types. *ES*_*k*_ is the ecological security index for the *k* th evaluation unit, where higher values correspond to greater ecological security levels, while lower values indicate reduced security.

##### Determination of evaluation criteria

To characterize the spatiotemporal variations of landscape ecological security within the study area, the ecological security index for each grid was categorized into five levels by means of the natural breaks method. The intervals for 1990, 2000, 2010, 2020, and 2023 were standardized based on the 1980 classification benchmarks (Table 2).

**Table 2:** Evaluation standard of landscape ecological security.

landscape ecological security index (*ES*_*k*_)	landscape ecological security level
0.0000 ≤ *ES*_*i*_ < 0.5204	Low safety
0.5204 ≤ *ES*_*i*_ < 0.6586	Lower safety
0.6586 ≤ *ES*_*i*_ < 0.7094	General safety
0.7094 ≤ *ES*_*i*_ < 0.7621	Safer
0.7621 ≤ *ES*_*i*_ < 1.0000	Safe

#### Sensitivity analysis of disturbance index weights

To evaluate the robustness of ecological security results to the choice of disturbance index weights, we tested three alternative weight schemes in addition to the baseline (*a* = 0.5, *b* = 0.3, *c* = 0.2): Scheme B (*a* = 0.4, *b* = 0.4, *c* = 0.2) and Scheme C (*a* = 0.6, *b* = 0.2, *c* = 0.2). For each scheme, *ES*_*k*_ values were recalculated across all 11,036 grid cells and six time periods. The spatial distribution of ecological security levels and the direction of temporal trends remained consistent across all three schemes. Spearman rank correlations between baseline *ES*_*k*_ values and those derived from Schemes B and C exceeded rs = 0.96 (p < 0.001) in all time periods, and the *Moran’s I* values varied by less than 0.008 across schemes. These results confirm that the conclusions of this study are robust to moderate variations in disturbance index weighting and are not an artifact of the specific weight choices adopted. Regarding the vulnerability weight structure, it is further noted that the linear normalization of ordinal vulnerability levels (Table 1) implicitly assumes equal spacing between vulnerability categories. This is a recognized simplification of the area-weighted summation approach (Eq. 8). The high rank correlation between *ES*_*k*_ values derived from alternative weight schemes (Spearman rs > 0.96) provides indirect evidence that moderate violations of this equal-spacing assumption do not substantially alter spatial patterns or temporal trends. Nonlinear transformation of vulnerability levels prior to normalization, or locally calibrated weights derived through Delphi expert elicitation under arid-zone conditions, represent methodological refinements recommended for future studies.

#### Spatial autocorrelation analysis

Spatial autocorrelation analysis is a method of measuring the spatial dependence of a geographic unit on its neighbours, enabling the identification of regional changes and spatial pattern characteristics^47,74^. In this study, we employed global (*Moran’s I*) and local (*LISA*) spatial autocorrelation analyses to evaluate the spatial clustering characteristics of landscape ecological security in the Kashgar region. Global *Moran’s I* reveals the overall spatial clustering pattern, whereas local *Moran’s I* identifies the specific cluster locations and types^75,76^. The calculation equation is as follows:

(1) Global spatial autocorrelation

9 $$Moran{\prime}s I =\frac{n\sum_{i=1}^{n}\sum_{j=1}^{n}\left({x}_{i}-\right.\left.\overline{x }\right)\left({x}_{j}\right.-\left.\overline{x }\right)}{\sum_{i=1}^{n}\sum_{j=1}^{n}{W}_{ij}{\left({x}_{i}\right.-\left.\overline{x }\right)}^{2}}$$

where, *n* is the total number of units; *x*_*i*_ and* x*_*j*_ are the observed values of units *i* and *j,* respectively; $\overline{x }$ is the mean of the variables; *W*_*ij*_ denotes the spatial weight between units *i* and *j* (usually, *W*_*ij*_ = 1 if the units are adjacent, otherwise *W*_*ij*_ = 0). The global *Moran’s I* ranges from [–1,1]. A value of *Moran’s I* > 0, < 0, = 0 indicates the clustered, dispersed and random distribution of the landscape ecological security in space, respectively.

(2) Local spatial auto-correlation

10 $${LISA}_{i} =\frac{\left({x}_{i}\right.-\left.\overline{x }\right)}{\sum_{i}\frac{{\left({x}_{i}\right.-\left.\overline{x }\right)}^{2}}{n}}\sum_{j}{W}_{ij}\left({x}_{j}\right.-\left.\overline{x }\right) i\ne j$$

where, *LISA*_*i*_ is not bounded by a fixed value range. A value of *LISA*_*i*_ > 0 suggests spatial clustering of similar values around a given spatial unit. Conversely, a value of *LISA*_*i*_ < 0 denotes spatial clustering of dissimilar values.

## Results

### Land use change analysis

#### Analysis of land use structural changes

**Fig. 2:**
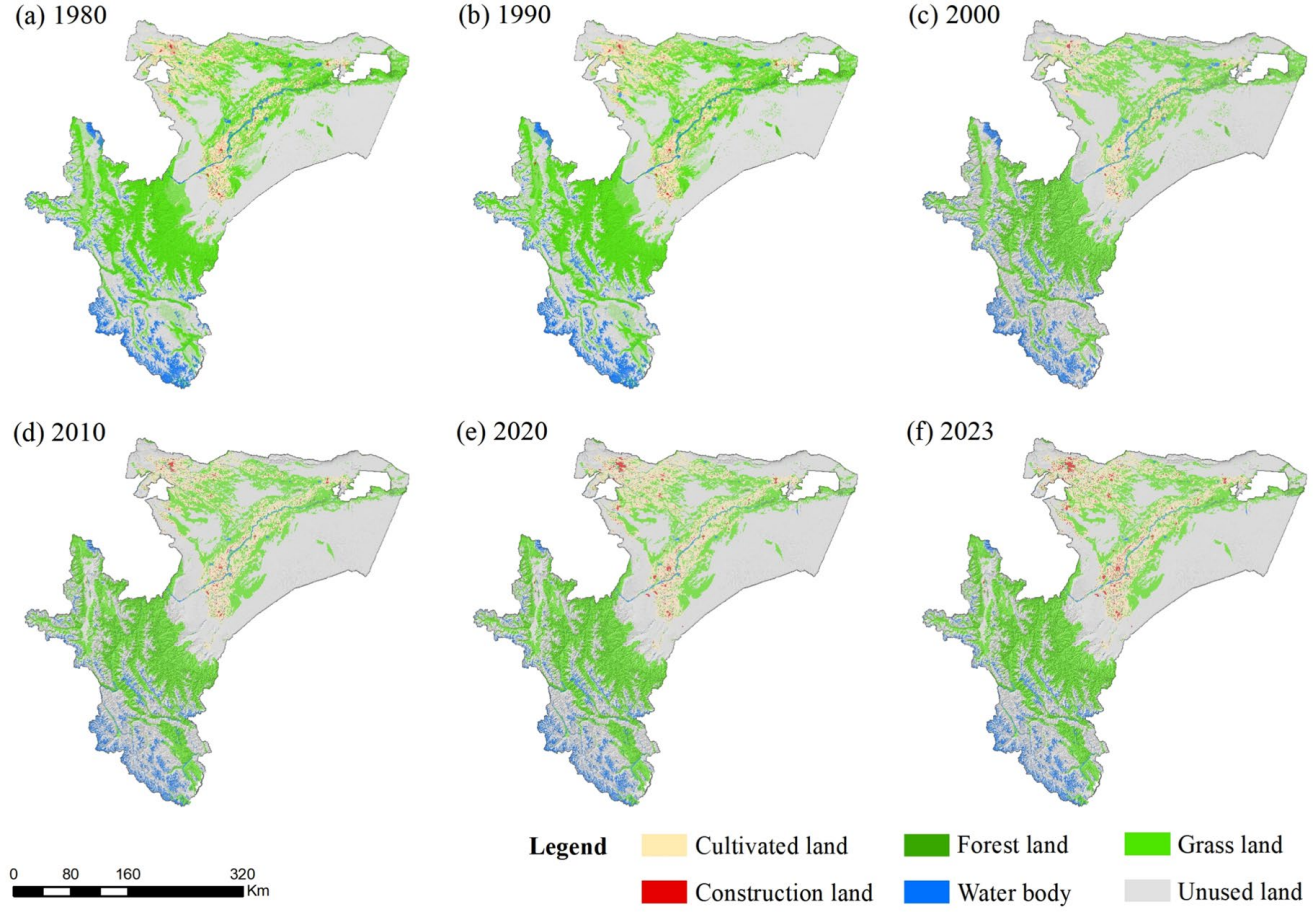
Spatial distribution of land use (**a**) 1980, (**b**) 1990, (**c**) 2000, (**d**) 2010, (**e**) 2020, (**f**) 2023. The map is generated with ArcGIS software (Version 10.8, https://www.esri.com).

Significant changes in land use were observed throughout the period spanning from 1980 to 2023 in the Kashgar region (Table 3 and Fig. 2). The predominant land use types in the Kashgar region are unused land, grass land and cultivated land, collectively accounting for 93.37% of the total study area.

**Table 3:** Land use structure change in the Kashgar region from 1980 to 2023.

Period	Land use type	Cultivated land	Forest land	Grass land	Water body	Construction land	Unused land	Total area
1980	Area/km^2^	8651.44	1612.20	34,327.71	5760.85	269.15	59,742.34	110,363.69
	Proportion/%	7.84	1.46	31.10	5.22	0.24	54.13	100.00
1990	Area/km^2^	8659.87	1643.81	34,381.79	5705.71	276.78	59,695.73	110,363.69
	Proportion/%	7.85	1.49	31.15	5.17	0.25	54.09	100.00
2000	Area/km^2^	8974.47	1678.60	34,296.18	5732.07	338.74	59,343.64	110,363.69
	Proportion/%	8.13	1.52	31.08	5.19	0.31	53.77	100.00
2010	Area/km^2^	11,023.26	1069.53	34,382.35	4970.74	618.35	58,299.46	110,363.69
	Proportion/%	9.99	0.97	31.15	4.50	0.56	52.82	100.00
2020	Area/km^2^	13,047.74	1050.35	32,564.44	4954.06	827.33	57,919.78	110,363.69
	Proportion/%	11.82	0.95	29.51	4.49	0.75	52.48	100.00
2023	Area/km^2^	12,323.22	1241.69	33,514.29	4828.95	1242.56	57,212.97	110,363.69
	Proportion/%	11.17	1.13	30.37	4.38	1.13	51.84	100.00
Variation (1980–1990)	Area/km^2^	+ 8.43	+ 31.62	+ 54.08	–55.14	+ 7.63	–46.61	0
Variation (1990–2000)	Area/km^2^	+ 314.60	+ 34.79	–85.61	+ 26.36	+ 61.95	–352.09	0
Variation (2000–2010)	Area/km^2^	+ 2048.78	–609.07	+ 86.17	–761.32	+ 279.62	–1044.18	0
Variation (2010–2020)	Area/km^2^	+ 2024.48	–19.18	–1817.91	–16.68	+ 208.97	–379.68	0
Variation (2020–2023)	Area/km^2^	–724.51	+ 191.34	+ 949.85	–125.11	+ 415.24	–706.81	0
Variation (1980–2023)	Area/km^2^	+ 3671.78	–370.51	–813.42	–931.89	+ 973.41	–2529.37	0

“ + ”means increased areas, “–” means decreased areas.

From 1980 to 2023, the total area of construction land increased by 973.41 km^2^, with an average annual increase of 22.64 km^2^/yr. Notably, the construction land expansion rate exhibited non-linear acceleration, with post-2010 annual increments (69.91 km^2^/yr during 2010–2023) exceeding pre-2010 rates (10.67 km^2^/yr during 1980–2010) by 6.5-fold. This inflection point coincides with intensified Belt and Road Initiative investments and Special Economic Zone establishment, suggesting policy-driven rather than organic urbanization dynamics. The most pronounced expansion occurred between 2020 and 2023, during which construction land increased by 415.24 km^2^ (138.41 km^2^/yr). In contrast, unused land demonstrated a continuous decline, with the most notable decrease from 59,742.34 km^2^ (54.13% of the total area) to 57,212.97 km^2^ (51.84%), a net reduction of 2.29%. Cultivated land expanded from 8651.44 km^2^ to 12,323.22 km^2^, with a net gain of 3671.78 km^2^, and an average annual increase of 85.39 km^2^/yr. This expansion occurred during 1980–1990, 1990–2000, 2000–2010, and 2010–2020, with the most substantial growth observed from 2000 to 2010, with an increase of 2048.78 km^2^ (204.88 km^2^/yr). However, cultivated land decreased between 2020 and 2023. Forest land area fluctuated throughout the study period: it increased from 1980 to 2000, declined between 2000 and 2020, and partially recovered from 2020 to 2023. Overall, forest land decreased by 370.51 km^2^ over the 43-year period, Specifically, increasing by 66.40 km^2^ from 1980 to 2000, then decreasing by 628.25 km^2^ from 2000 to 2020, before rebounding by 191.34 km^2^ between 2020 and 2023. Grass land showed periodic volatility, characterised by alternating periods of increase and decrease. It increased during 1980–1990, 2000–2010, and 2020–2023, and decreased during 1990–2000 and 2010–2020. During the study period, grass land decreased by 813.42 km^2^, with an average annual reduction of 18.92 km^2^/yr. Similarly, water area exhibited pronounced fluctuations, decreasing from 1980 to 1990, then increasing slightly by 2000, before declining markedly from 2000 to 2023. Overall, water bodies decreased by 931.89 km^2^, with an average annual reduction of 21.67 km^2^/yr.

#### Changes in land use dynamic degree and intensity

**Fig. 3:**
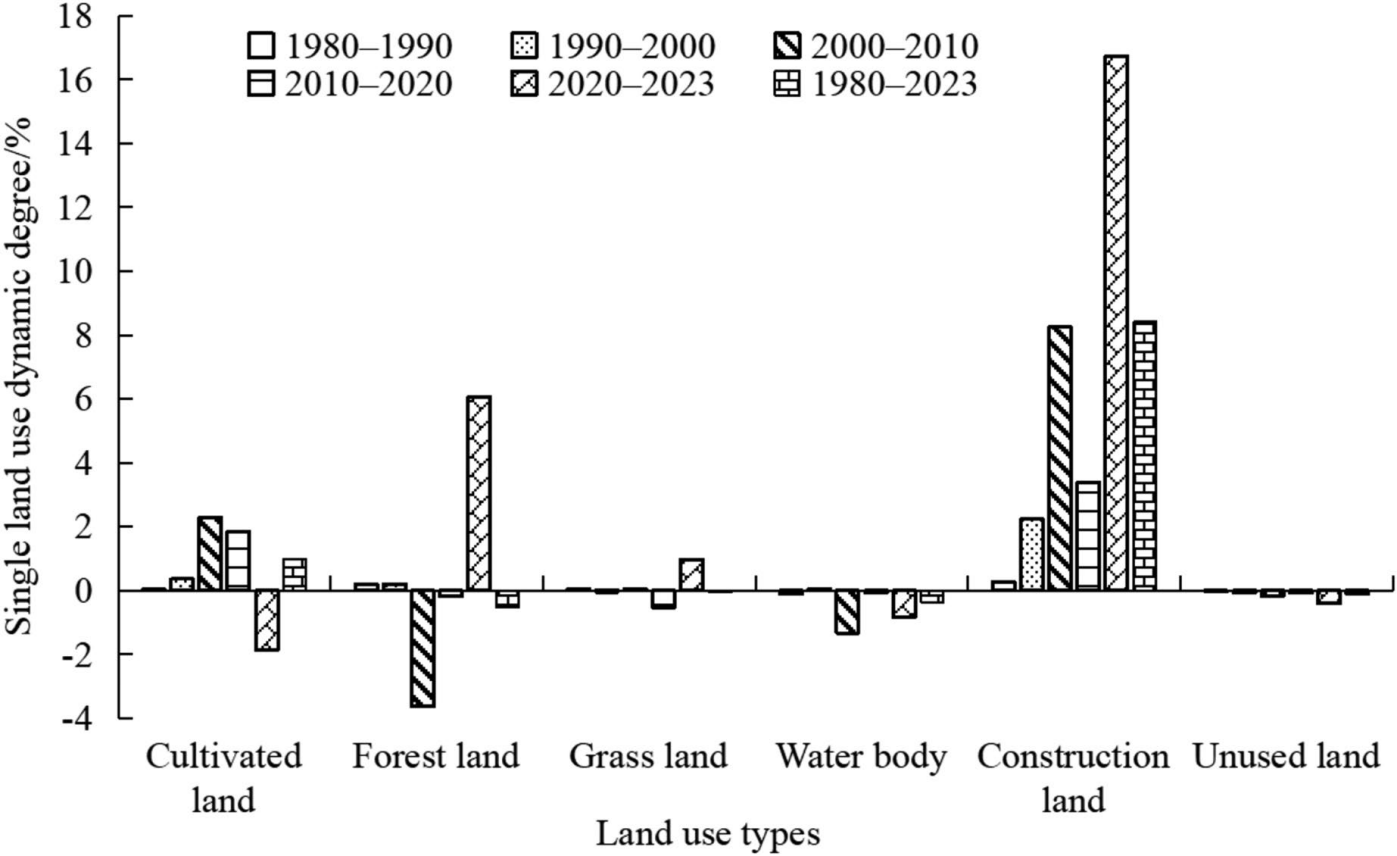
Land use dynamic degree in Kashgar region from 1980 to 2023.

Land use transformation rates quantify landscape reorganization intensity. Based on land use data from 1980 to 2023, this study quantified both the single and integrated land use dynamic degree for various land use types, with results presented in Table 4 and Fig. 3.

**Table 4:** Changes in land use dynamic degree in the Kashgar region from 1980 to 2023.

Dynamic degree	Single dynamic degree (K/%)						Integrated dynamic degree (LC/%)
Period	Cultivated land	Forest land	Grass land	Water body	Construction land	Unused land	
1980–1990	0.01	0.20	0.02	–0.10	0.28	–0.01	0.02
1990–2000	0.36	0.21	–0.02	0.05	2.24	–0.06	0.29
2000–2010	2.28	–3.63	0.03	–1.33	8.25	–0.18	1.20
2010–2020	1.84	–0.18	–0.53	–0.03	3.38	–0.07	0.15
2020–2023	–1.85	6.07	0.97	–0.84	16.73	–0.41	0.31
1980–2023	0.99	–0.53	–0.06	–0.38	8.41	–0.10	0.31

As shown in Table 4, the integrated land use dynamic degree in the Kashgar region exhibited a overall fluctuating trend during the 1980–2023. From 2000 to 2010 recorded the highest land use dynamic degree at 1.20%, followed by a marked deceleration to 0.15% during 2010–2020, before increasing again to 0.31% between 2020 and 2023. These fluctuations indicate that internal structural changes in land use have been relatively complex and frequent over the past 43 years, with the activity levels of transitions between different types being uneven.

Regarding single land use dynamic degree, construction land exhibited the most pronounced expansion, with rates accelerating markedly across periods. It should be noted that the 2020–2023 interval spans only three years compared to the ten-year intervals of other periods; the annualized dynamic degree of 16.73% therefore reflects both genuine acceleration in construction activity and a methodological amplification effect inherent in shorter observation windows. The absolute area increase of 415.24 km^2^ during this three-year period (138.41 km^2^/yr) provides a scale-independent confirmation of accelerated expansion, highlighting a phase of rapid urbanization and economic development. Cultivated land displayed a more complex trend: It expanded continuously from 1980 to 2020, with the fastest growth during 2000–2010 (2.28%), but underwent a sharp reversal between 2020 and 2023, declining at an annual rate of –1.85%. Forest land also experienced substantial fluctuations. After a period of slow growth, it declined significantly during 2000–2010 (–3.63%), followed by a strong recovery in the latest period (2020–2023), reflecting the results of large-scale afforestation and ecological restoration policies. Grass land and water bodies remained relatively stable overall but showed some periodic fluctuations. Grass land declined notable during 2010–2020 (–0.53%), while water bodies experienced significant loss in 2000–2010 (–1.33%). Unused land consistently decreased throughout the study period at an average annual rate of 0.11%, indicating ongoing conversion of marginal lands to other uses.

On the whole, the evolving land use dynamics in the Kashgar region, characterised by the decline of natural landscapes such as forest and grass land, alongside the expansion of construction land underscore the profound impacts of economic development, population growth, and urban expansion.

**Fig. 4:**
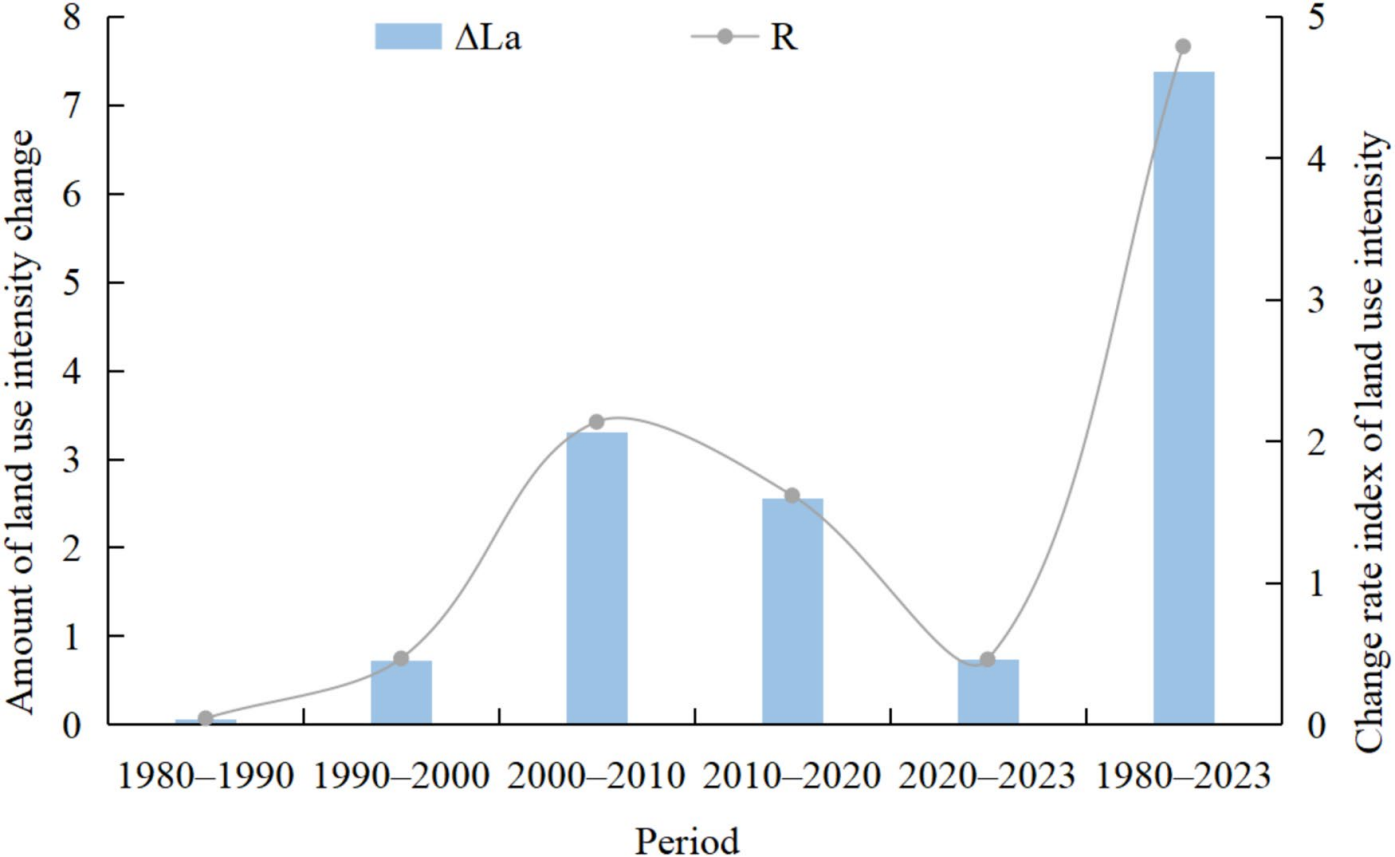
The comprehensive index and change index of land use intensity.

As shown in Table 5, increased from 154.19 in 1980 to 161.58 in 2023 (a net change of 7.38 units within the theoretical range of 100–400, representing a 2.5% relative increase), reflecting a moderate but consistent intensification of land development over the 43-year period. The first two decades (1980–2000) were characterised by slow growth, with the amounts of land use intensity change (Δ*La*) values of 0.06 and 0.72, and correspondingly low change rates (*R* of 0.04% and 0.46%, respectively). In contrast, the period from 2000 to 2020 exhibited a marked intensification of land use. Both the Δ*La* and *R* peaked during 2000–2010, with Δ*La* reaching 3.31 and *R* reaching 2.14%. From 2020–2023, the change rate index (*R* = 0.46%) returned to a level similar to that of 1990–2000. Overall, the comprehensive index of land use intensity (*La*) in the study area is evolving toward a normal state. Moreover, both Δ*La* and *R* remained greater than 0 throughout the entire study period (Fig. 4), indicating that land use in the region is still in a developmental phase. This further suggests potential for optimizing land use efficiency, and that the land use structure could be effectively improved through policy interventions, regulatory measures, and the establishment of relevant rules and regulations.

**Table 5:** The comprehensive index and change index of land use intensity.

Period	land use intensity		
	*La*	∆*La*	*R*
1980	154.19	–	–
1990	154.26	–	–
2000	154.97	–	–
2010	158.28	–	–
2020	160.84	–	–
2023	161.58	–	–
1980–1990	–	0.06	0.04
1990–2000	–	0.72	0.46
2000–2010	–	3.31	2.14
2010–2020	–	2.56	1.62
2020–2023	–	0.74	0.46
1980–2023	–	7.38	4.79

#### Spatiotemporal characteristics of land use

**Fig. 5:**
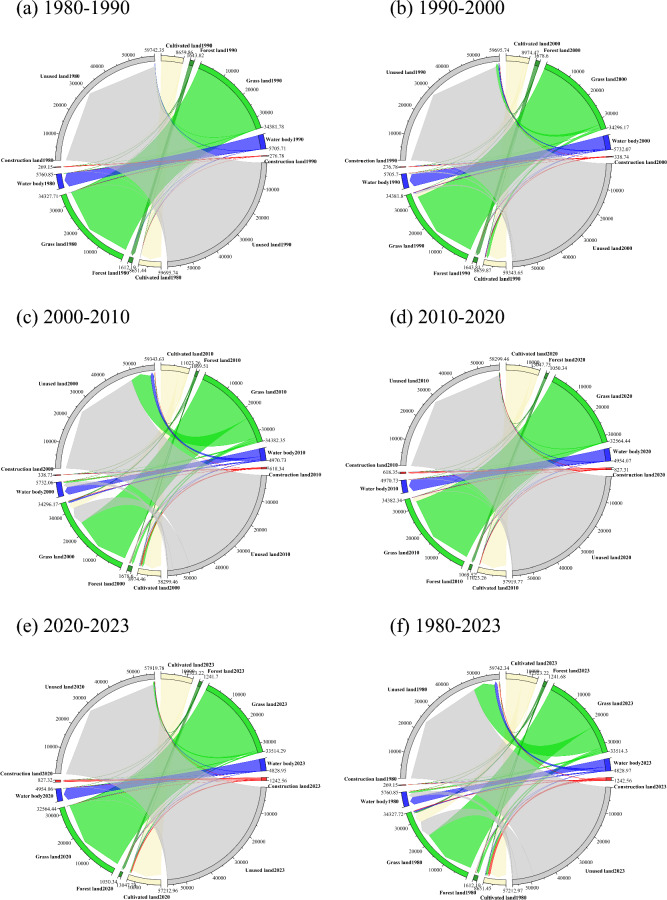
Transfer direction between different landscape types (**a**) 1980 to 1990, (**b**) 1990 to 2000, (**c**) 2000 to 2010, (**d**) 2010 to 2020, (**e**) 2020 to 2023, (**f**) 1980 to 2023 (unit:km^2^).

The land use transfer matrix from 1980 to 2023 reveals the transition relationships among different land use categories (Table 6 and Fig. 5).

**Table 6:** Landscape type transfer matrix of Kashgar region from 1980 to 2023 (unit:km^2^).

Period	Land use type	Cultivated land	Forest land	Grass land	Water body	Construction land	Unused land	Total
1980–1990	Cultivated land	8620.16	0.39	12.16	13.04	0.97	4.72	8651.44
	Forest land	0.05	1610.45	0.29	1.23	0.00	0.17	1612.20
	Grass land	1.45	0.28	34,262.28	41.59	7.66	14.45	34,327.71
	Water body	31.63	31.82	85.22	5600.25	0.16	11.77	5760.85
	Construction land	1.13	0.02	0.01	0.00	267.99	0.00	269.15
	Unused land	5.44	0.86	21.82	49.60	0.00	59,664.63	59,742.34
	Total in 1990	8659.87	1643.81	34,381.79	5705.71	276.78	59,695.73	110,363.69
1990–2000	Cultivated land	7153.15	94.88	1003.58	49.10	160.35	198.81	8659.87
	Forest land	84.64	1522.74	14.63	11.19	0.52	10.09	1643.81
	Grass land	1429.30	34.92	32,036.93	174.22	22.58	683.85	34,381.79
	Water body	21.32	0.91	35.82	5134.08	0.01	513.56	5705.71
	Construction land	116.93	2.49	7.93	0.41	148.28	0.74	276.78
	Unused land	169.13	22.66	1197.28	363.07	7.00	57,936.60	59,695.73
	Total in 2000	8974.47	1678.60	34,296.18	5732.07	338.74	59,343.64	110,363.69
2000–2010	Cultivated land	7210.57	335.61	754.75	70.48	409.49	193.56	8974.47
	Forest land	311.55	278.13	818.91	41.80	13.17	215.04	1678.60
	Grass land	2659.85	370.39	23,845.98	481.74	57.02	6881.19	34,296.18
	Water body	132.33	10.89	618.19	3162.38	5.11	1803.16	5732.07
	Construction land	188.11	17.58	10.92	0.69	114.21	7.22	338.74
	Unused land	520.85	56.91	8333.60	1213.64	19.34	49,199.29	59,343.64
	Total in 2010	11,023.26	1069.53	34,382.35	4970.74	618.35	58,299.46	110,363.69
2010–2020	Cultivated land	10,770.90	18.89	88.18	10.88	115.22	19.19	11,023.26
	Forest land	32.82	1003.74	17.09	2.44	4.57	8.86	1069.53
	Grass land	1876.10	16.31	32,285.64	32.33	49.58	122.38	34,382.35
	Water body	15.40	1.96	46.76	4795.31	0.53	110.77	4970.74
	Construction land	28.18	0.90	0.44	0.56	587.87	0.40	618.35
	Unused land	324.33	8.54	126.33	112.55	69.54	57,658.17	58,299.46
	Total in 2020	13,047.74	1050.35	32,564.44	4954.06	827.33	57,919.78	110,363.69
2020–2023	Cultivated land	12,271.91	20.30	357.48	7.27	372.42	18.36	13,047.74
	Forest land	14.93	976.72	43.91	1.05	10.53	3.20	1050.35
	Grass land	9.46	129.15	32,402.37	1.66	8.22	13.58	32,564.44
	Water body	7.86	11.78	113.40	4740.38	3.05	77.59	4954.06
	Construction land	0.14	0.60	0.18	0.01	826.39	0.00	827.33
	Unused land	18.92	103.15	596.95	78.58	21.95	57,100.23	57,919.78
	Total in 2023	12,323.22	1241.69	33,514.29	4828.95	1242.56	57,212.97	110,363.69
1980–2023	Cultivated land	6571.52	309.02	693.00	70.48	851.68	155.75	8651.44
	Forest land	288.23	308.42	775.06	36.49	20.32	183.67	1612.20
	Grass land	4206.03	524.26	22,457.34	447.34	174.69	6518.06	34,327.71
	Water body	182.32	9.51	654.86	3119.36	4.76	1790.04	5760.85
	Construction land	162.33	12.16	8.54	1.33	81.36	3.43	269.15
	Unused land	912.79	78.31	8925.50	1153.97	109.75	48,562.02	59,742.34
	Total in 2023	12,323.22	1241.69	33,514.29	4828.95	1242.56	57,212.97	110,363.69

From 1980 to 1990, the expansion of cultivated land and grass land was primarily sourced from water bodies and unused land, accounting for 31.63 km^2^ and 5.44 km^2^ transferred to cultivated land, and 85.22 km^2^ and 21.82 km^2^ transferred to grass land, respectively. Newly increased forest land was mainly converted from water bodies, with a transferred area of 31.82 km^2^, constituting the dominant source of forest land gain. Construction land expansion was predominantly derived from grass land, with 7.66 km^2^ transferred, representing the dominant source of construction land growth. In terms of transition directions, water bodies were transformed into grass land, forest land, and cultivated land, while unused land was mainly converted into water bodies and grass land. Between 1990 and 2000, cultivated land, forest land and construction land continued to expand. The increase in cultivated land was primarily derived from grass land (1429.30 km^2^) and unused land (520.85 km^2^). The increase in forest land was mainly due to cultivated land (94.88 km^2^) and grass land (34.92 km^2^). Construction land expansion was sourced from cultivated land (160.35 km^2^). Meanwhile, active bidirectional conversion persisted between grass land and unused land, with 683.85 km^2^ of grass land converted to unused land and 1197.28 km^2^ of unused land converted to grass land. These bidirectional transitions reveal competing ecological processes: the conversion of unused land and grassland to cultivated land (net flux: + 3671.78 km^2^) indicates agricultural frontier expansion driven by food security imperatives, while the grassland-unused land exchange (net positive: + 513.43 km^2^) suggests that ecological restoration programs partially offset degradation pressures. The asymmetric transfer dynamics—with construction land functioning as an irreversible sink and unused land as a persistent source—establish a unidirectional landscape evolution trajectory toward anthropogenic dominance. The period from 2000 to 2010 was characterised by large-scale expansion of cultivated land and a concurrent substantial decrease in unused land. Grass land remained the primary source of this expansion, with a transferred area of 2659.85 km^2^. Unused land was primarily converted into grass land and water bodies, with transition areas of 8333.60 km^2^ and 1213.64 km^2^, respectively, suggesting that ecological restoration policies during this period may have contributed to the conversion of unused land to grass land. During the 2010–2020 period, cultivated land continued to expand, predominantly sourced from grass land (1876.10 km^2^) and unused land (324.33 km^2^). Meanwhile, forest land was mainly converted to cultivated land, grass land, and unused land, suggesting issues of forest degradation and deforestation for agricultural use. Construction land expansion was mainly derived from cultivated land (115.22 km^2^), reflecting urbanization’s encroachment on agricultural land. From 2020 to 2023, cultivated land area slightly decreased, while forest land and grass land areas rebounded significantly. Of which, the conversion of cultivated land to construction land and grass land accounted for 48% and 46.08% of the total cultivated land transfer area, respectively. Forest land gains mainly came from grass land (129.15 km^2^) and unused land (103.15 km^2^), while grass land expansion primarily originated from unused land (596.95 km^2^) and cultivated land (357.48 km^2^). This indicates that ecological conservation policies, such as reforestation and grass land restoration, may have contributed to the partial recovery of forest and grass land ecosystems, primarily through the utilization of unused land and the strategic conversion of marginal farmland.

In summary, over the past 43 years, construction land in the Kashgar region consistently maintained a unidirectional inflow imbalance, while, unused land exhibited a persistent unidirectional outflow imbalance. Cultivated land, forest land, grass land and water bodies engaged in bidirectional transitions with other land use types. The main transformation pathways included: cultivated land to construction land and grass land; forest land to grass land and cultivated land; grass land to unused land and cultivated land; water bodies to unused land and grass land; and unused land to grass land and water bodies.

### Landscape pattern change analysis

#### Analysis of landscape pattern indices at class level

**Fig. 6:**
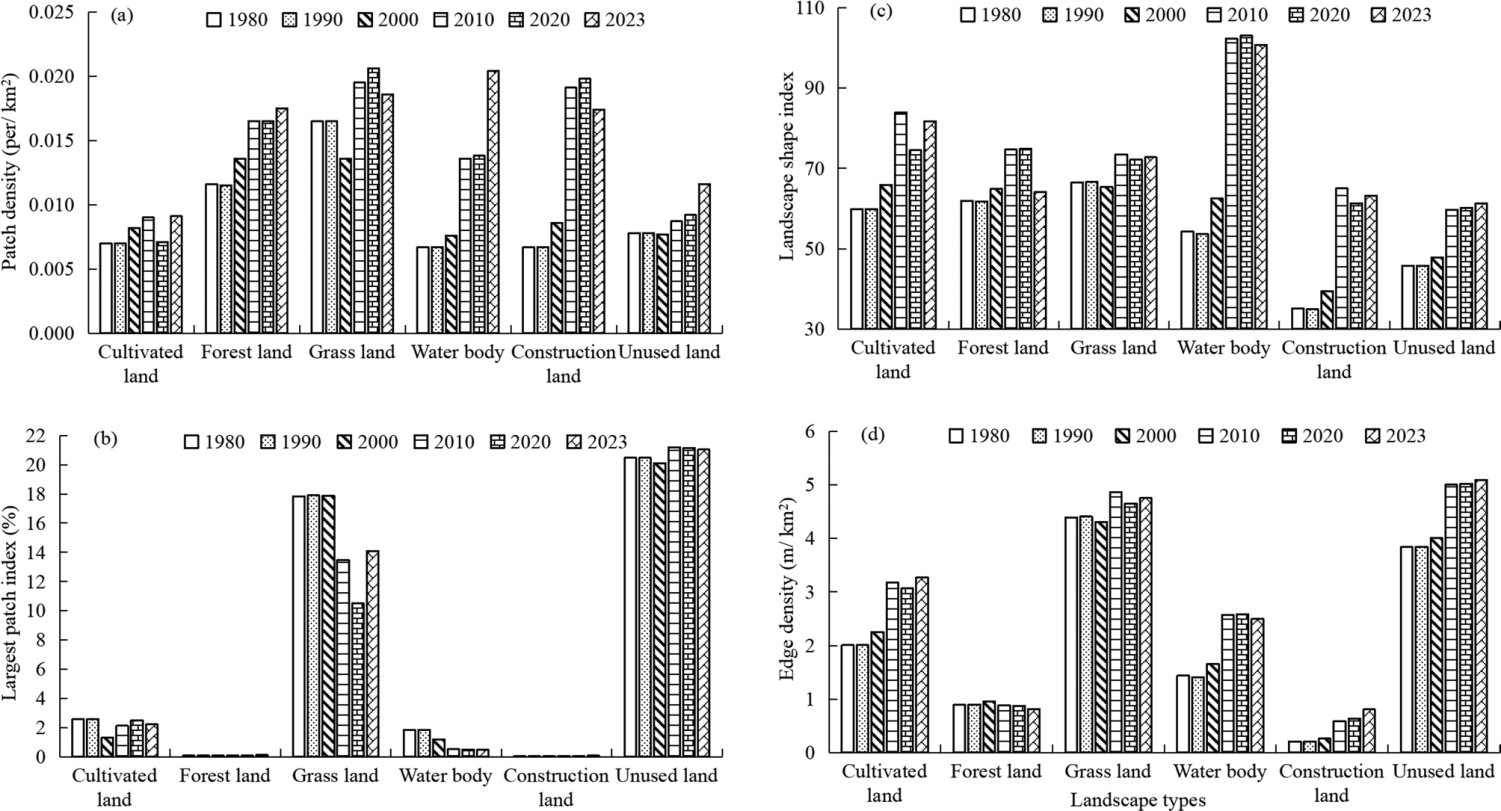
Variation of landscape pattern indices (**a**) PD, (**b**) LPI, (**c**) LSI, (**d**) ED at class level in the study area from 1980 to 2023.

As can be seen in Fig. 6, patch density (PD) of all landscape types in the Kashgar region remained relatively low during 1980–2023 but demonstrated an overall increasing trend, indicating varying degrees of disturbance across landscape types. The changes in water bodies were particularly notable, primarily attributable to the region’s distinctive climatic regime where evaporation substantially exceeds precipitation^77^. The scarcity of water resources has rendered numerous water bodies susceptible to conversion into unused land and grass land, resulting in progressively fragmented spatial distribution of water bodies. The largest patch index (LPI) is defined as the composition of the largest patch in the landscape type, and serves as an indicator of disturbance intensity from anthropogenic activities. In the Kashgar region, LPI dominated by unused land, grass land and cultivated land. The grass land LPI exhibited a gradual decline from 17.844% in 1980 to 14.099% in 2023, indicating intermittent fragmentation and encroachment of core grass land patches alongside a moderate reduction in ecological dominance. Concurrently, LPI of unused land maintained consistently high, suggesting the fundamental structure remained largely intact. The overall declining trend in water body LPI (from 1.855 to 0.482) signifies the degradation and fragmentation of natural ecological elements driven by a combination of climatic factors (predominantly intensified evaporation under warming temperatures) and, to a lesser extent, anthropogenic water use. The landscape shape index (LSI) for all landscape types generally exhibited a rising trend, with the most substantial changes observed in water bodies, construction land, and cultivated land (increases of 46.40, 28.04, and 21.84, respectively). This trend indicates an increasing irregularity of landscape patches and more complex landscape shapes resulting from anthropogenic disturbances. Except for forest land, which showed decreased edge density (ED), the ED of other landscape types exhibited an increase, with the most pronounced changes occurring in cultivated land, unused land, and water bodies.

#### Analysis of landscape pattern indices at landscape level

**Fig. 7:**
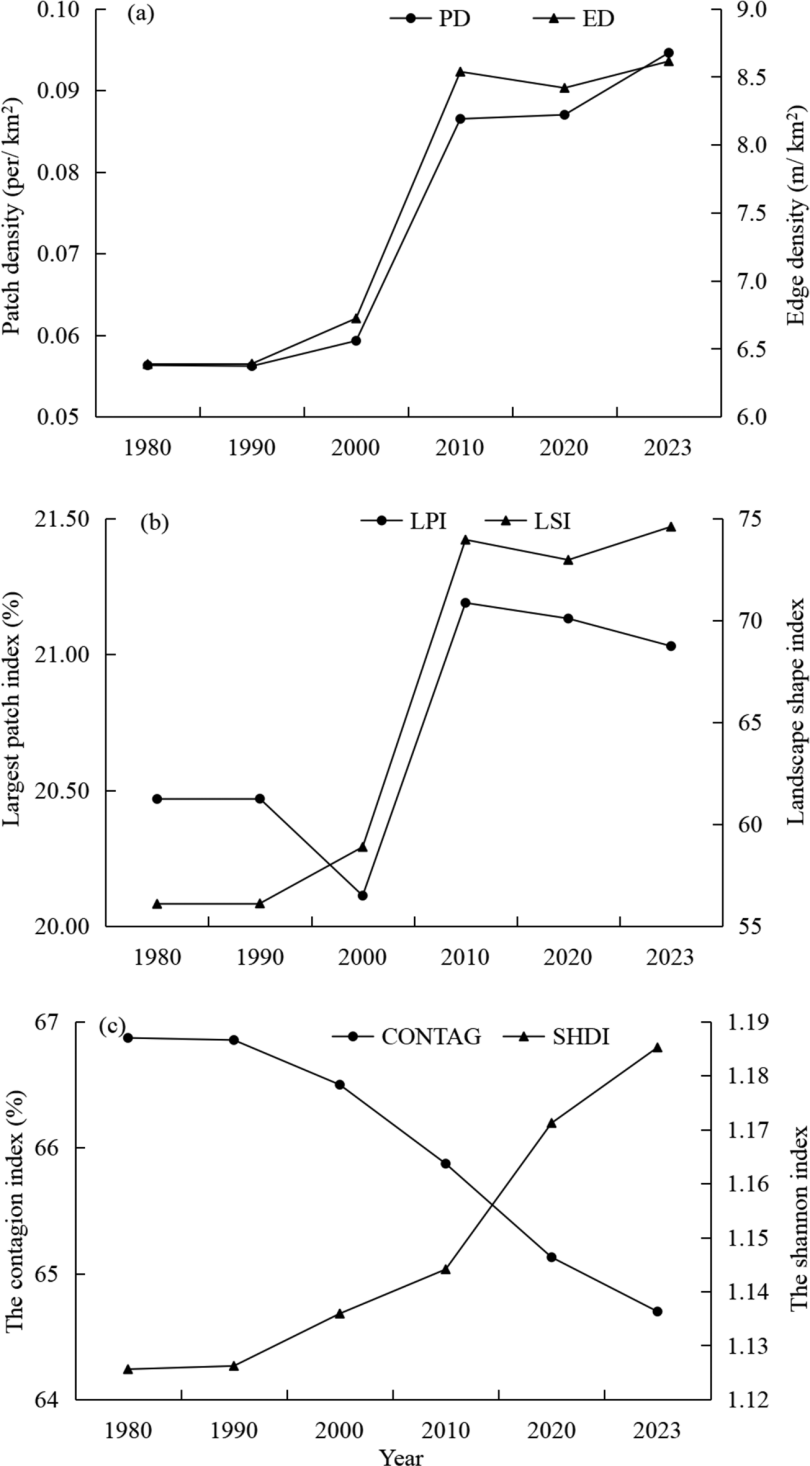
Temporal trends of landscape pattern indices (**a**) PD & ED, (**b**) LPI & LSI, (**c**) CONTAG & SHDI from 1980 to 2023.

As shown in Table 7, the landscape pattern indices in the Kashgar region, including patch density (PD), largest patch index (LPI), landscape shape index (LSI), edge density (ED), and shannon’s diversity index (SHDI), generally increased from 1980 to 2023, while the contagion index (CONTAG) demonstrated a decreasing trend over the same period. Specifically, PD increased from 0.056 to 0.095 per km^2^, indicating intensified landscape fragmentation and a more dispersed spatial distribution of patches. LPI increased from 20.468 in 1980 to a peak of 21.189 in 2010, then declined gradually to 21.030 in 2023, suggesting that the dominance of major patch types strengthened over the first three decades before partially receding. LSI increased from 56.097 to 74.590, suggesting more complex and irregular patch shapes as well as greater diversity of shape types. The significant increase in edge density (Fig. 7) was driven by the combined effects of rising patch density and shape complexity. SHDI also increased by 5.29%, signaling richer and more diversified landscape types. In contrast, CONTAG declined by 3.25%, indicating reduced landscape connectivity, heightened fragmentation, and diminished resistance to external disturbances.

**Table 7:** Changes in landscape pattern indices in Kashgar region from 1980 to 2023.

Year	PD	LPI	LSI	ED	CONTAG	SHDI
1980	0.056	20.468	56.097	6.387	66.870	1.126
1990	0.056	20.469	56.117	6.389	66.853	1.126
2000	0.059	20.113	58.889	6.723	66.498	1.136
2010	0.087	21.189	73.952	8.537	65.872	1.144
2020	0.087	21.132	72.961	8.418	65.129	1.171
2023	0.095	21.030	74.590	8.614	64.698	1.185

In summary, intensified human activities, including urbanization and economic development—have driven the conversion of ecological and natural landscapes into artificial landscapes. These landscape pattern changes suggest potential pressures on ecosystem connectivity and habitat integrity, which may have implications for ecological functions and regional sustainable development. Therefore, it is essential to implement effective measures to mitigate human-induced disturbances on landscape patterns and strengthen the protection of natural ecosystems through targeted land use planning and policy regulation.

### Evolution of landscape ecological security pattern

#### Landscape ecological security analysis

**Fig. 8:**
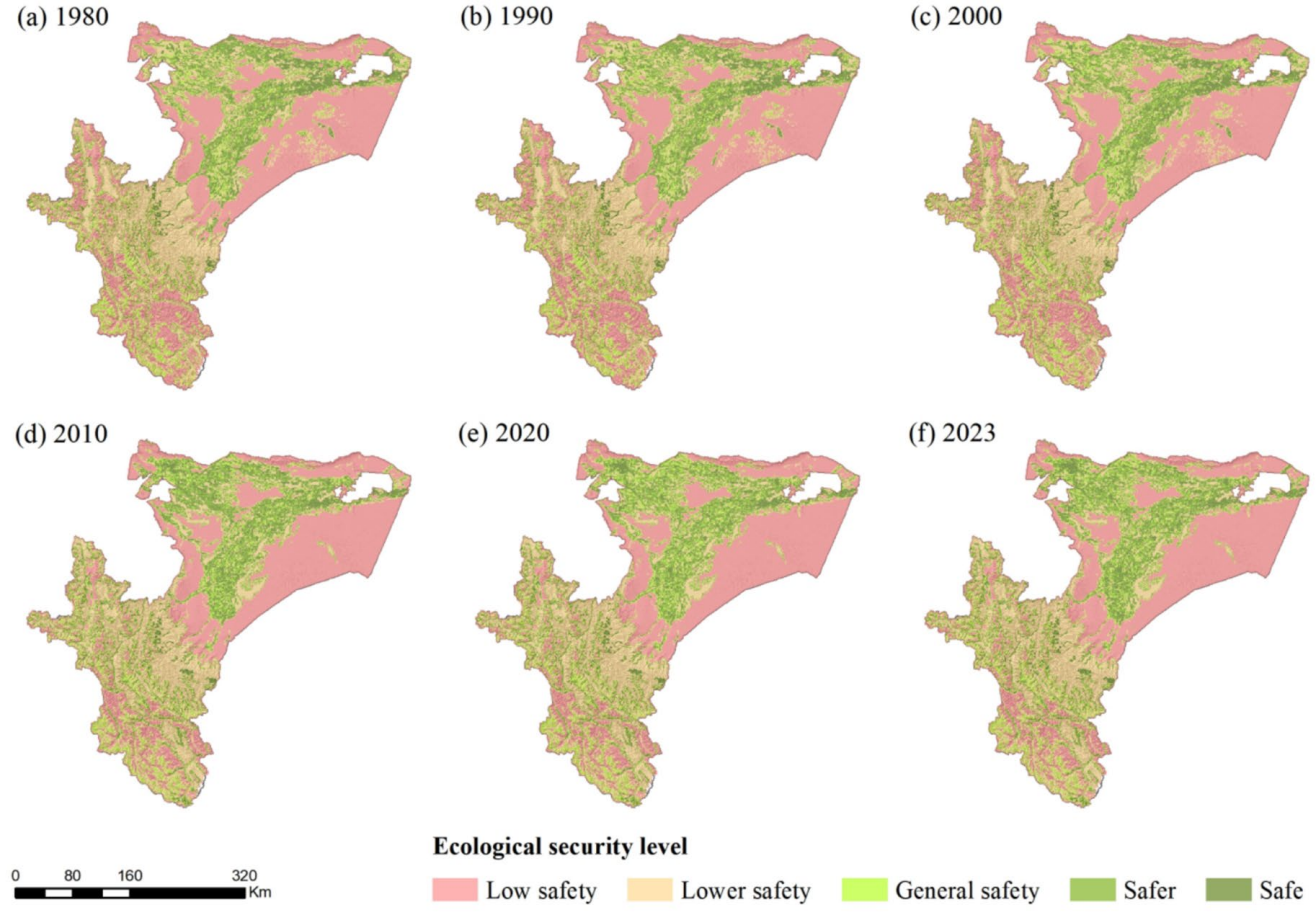
Spatio-temporal changes of landscape ecological security pattern in Kashgar region (**a**) 1980, (**b**) 1990, (**c**) 2000, (**d**) 2010, (**e**) 2020, (**f**) 2023. The map is generated with ArcGIS software (Version 10.8, https://www.esri.com).

From 1980 to 2023, the landscape ecological security level in the study area exhibited a pattern of initial improvement, followed by decline and subsequent recovery. Overall, the region was predominantly characterised by low- and lower-safety levels, reflecting a generally fragile ecological baseline. Spatially, higher security levels were observed in the southeastern and northwestern parts, while lower levels were concentrated along the eastern periphery. Nevertheless, landscape ecological security showed an overall improving trend (Table 8 and Fig. 8). Specifically, in 1980, low- and lower-safety zones accounted for 71.35% of the study area, primarily distributed in marginal regions such as Makit and Maralbexi County in the east and Konasheher County in the northwest. These areas generally exhibited characteristics of interspersed land use types, predominantly unused land and construction land. In contrast, safer and safe zones comprised 20.15% of the area, concentrated in southeastern regions characterised by forest and grass land coverage and relatively intact land use structures. Between 1990 and 2000, the spatial pattern of ecological security remained generally stable, with persistently high levels in the southeast and low levels in the eastern and northwestern fringes. A gradual overall improvement was also observed during this period. Specifically, the proportion of areas classified as general safety and below declined from 79.80% in 1990 to 79.67% in 2000, while the proportion of safer and safe zones increased from 20.20% to 20.33%. By 2010, landscape ecological security levels improved significantly, with the proportion of safer and safe zones reaching 22.30%. The area underwent an expansion of 2179.50 km^2^ compared to 2000, with the most notable spatial improvements occurring in the northwest (Fig. 8). However, ecological security declined between 2010 and 2020, as the proportion of safer and safe zones decreased to 21.78%, a reduction of 574.40 km^2^ compared to 2010. Concurrently, the proportion of low- and lower-safety zones decreased to 68.01%. By 2023, ecological security levels had recovered, with the combined proportion of safer and safe zones rising to 23.02%, while low- and lower-safety zones decreased to 67.51% (Table 8). This trend is broadly consistent with ongoing ecological restoration efforts.

**Table 8:** Area and proportion change of different ecological security grades in Kashgar region from 1980 to 2023.

Period	Ecological level	Low safety	Lower safety	General safety	Safer	Safe
1980	Area/km^2^	45,694.66	33,047.68	9379.48	15,956.02	6285.83
	Proportion/%	41.40	29.94	8.50	14.46	5.70
1990	Area/km^2^	45,684.53	33,034.81	9348.48	15,992.02	6303.83
	Proportion/%	41.39	29.93	8.47	14.49	5.71
2000	Area/km^2^	44,750.51	33,352.61	9823.86	16,229.87	6206.82
	Proportion/%	40.55	30.22	8.90	14.71	5.62
2010	Area/km^2^	42,680.28	33,026.72	10,040.49	17,034.46	7581.72
	Proportion/%	38.67	29.93	9.10	15.43	6.87
2020	Area/km^2^	42,305.73	32,747.65	11,270.52	17,583.98	6455.80
	Proportion/%	38.33	29.67	10.21	15.93	5.85
2023	Area/km^2^	41,918.32	32,583.22	10,461.66	18,239.65	7160.82
	Proportion/%	37.98	29.52	9.48	16.53	6.49

#### Spatial autocorrelation analysis of landscape ecological security

**Fig. 9:**
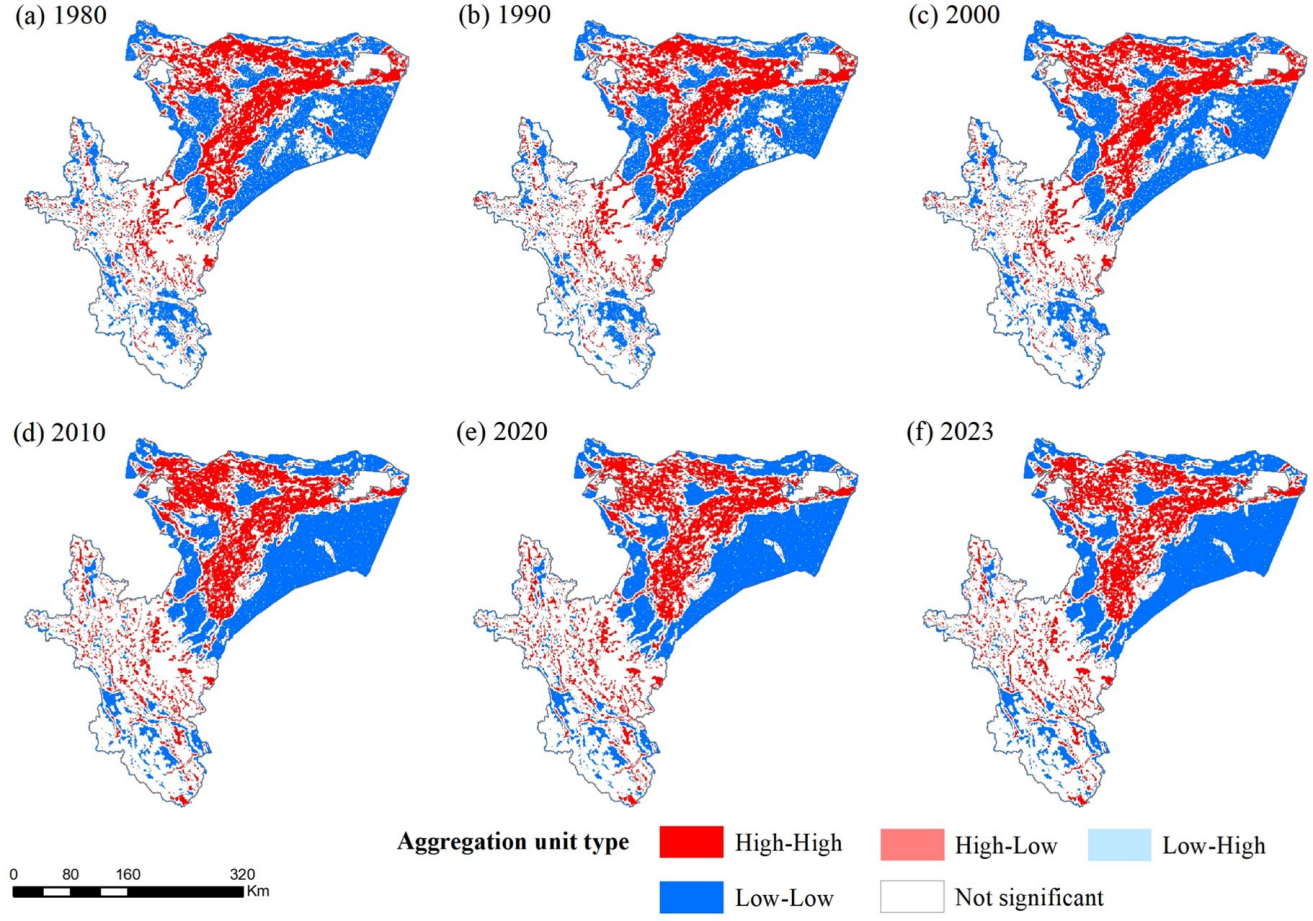
The LISA cluster map of landscape ecological security in the Kashgar region (**a**) 1980, (**b**) 1990, (**c**) 2000, (**d**) 2010, (**e**) 2020, (**f**) 2023. The map is generated with ArcGIS software (Version 10.8, https://www.esri.com).

From 1980 to 2023, the global *Moran’s I* values for the landscape ecological security index in the Kashgar region across six time periods were 0.7842, 0.7846, 0.7850, 0.8057, 0.8001, and 0.8044, respectively (P < 0.001, Z > 2.58). All *Moran’s I* values exceeded 0.7, indicating a persistently high and broadly stable positive spatial correlation in landscape ecological security across the study area, with minor fluctuations between periods (range: 0.7842–0.8057). The spatial distribution was non-random and demonstrated significant spatial dependence and clustering effects. The local *LISA* clustering map (Fig. 9) further revealed that the spatial distribution was dominated by concentrated and stable "high-high", "low-low" clusters. In contrast, "low–high", "high-low" clusters were more dispersed characteristics. Specifically, "high-high" clusters were primarily distributed in the Yarkant River and Kashgar oases, whereas "low-low" clusters were concentrated along the eastern marginal zone adjacent to the Taklamakan Desert, encompassing areas such as Makit and Maralbexi County. These areas represent typical ecological transition zones that exhibit high sensitivity to environmental changes.

From a local evolution perspective, the "high-high" clusters in the southeastern and northwestern parts of the study area gradually expanded in area from 1980 to 2023, with their spatial distribution becoming more concentrated, suggesting a spatial consolidation of high-security patches over the study period, though absolute security levels showed inter-period fluctuations including a decline during 2010–2020. Although the area of "low–low" clusters along the eastern periphery decreased, their spatial distribution became more concentrated, suggesting that despite overall regional ecological improvement, this area remains vulnerable to potential ecological degradation.

The results demonstrate that the southeastern and northwestern parts of the Kashgar region, which are concentrated areas of forest and grass land, primarily function as ecological barriers. It is essential to continuously consolidate their ecological functions in the future. In contrast, the eastern periphery characterised by an oasis-desert transition and extensive unused land—exhibits highly fragile and unstable ecosystems. Therefore, future efforts should prioritize ecological construction and protection in this area through diversified governance strategies. In "high–high" clusters, the focus should be on optimizing ecological patterns, strengthening water source protection, maintaining forest and grass land ecosystem functions, and promoting ecological agricultural models. In "low-low" clusters, precise ecological prevention and control measures should be implemented, including enhanced protection and restoration efforts, desertification control projects, and intelligent water resource management. Based on the preliminary monitoring reference of Δ*La* ≈ 2.5–3.5 per decade (with 3.0 as the central management reference pending cross-regional validation), the following management responses are provisionally suggested. Should Δ*La* approach or exceed 3.0 in any given decade, early intervention measures may be considered, including freezing new construction approvals in ecologically sensitive zones, mandatory ecological compensation payments, and emergency restoration funding allocation. When Δ*La* falls between 2.0 and 3.0, preventive measures should be activated, such as enhanced land use auditing at county level and accelerated implementation of the Grain for Green program. When Δ*La* remains below 2.0, routine monitoring and maintenance of existing ecological restoration programs is sufficient. These measures are expected to effectively alleviate ecological pressures, improve ecosystem resilience and sustainability, and comprehensively enhance the regional ecological security.

## Discussion

### Selecting and analyzing landscape pattern indices

In selecting landscape pattern indices, comprehensive coverage of all dimensions is unnecessary. Given the functional redundancy among numerous landscape indices, a limited number of representative indices are sufficient to adequately address the requirements of landscape pattern analysis and effectively captures the landscape characteristics of the study area. To avoid information redundancy caused by highly correlated indices, this study selected six landscape pattern indices representing six dimensions—area, density, edge, shape, diversity, and aggregation/dispersion—to quantify landscape fragmentation, shape complexity, and diversity. This study further conducted analyses at both class and landscape levels, and characterised pattern changes for landscape types and the overall landscape across different periods.

At the class level, LPI and PD were selected to characterize landscape features from the perspectives of area-based dominance and fragmentation, respectively. LPI reflects the concentration of dominant patches within the landscape, while PD indicates the degree of landscape fragmentation. Additionally, LSI and ED were adopted to describe the complexity of patch boundaries from a shape perspective. LSI measures patch aggregation and dispersion, whereas ED represents the total edge length per unit area. The combined use of these indices avoids informational redundancy while capturing complementary aspects of landscape patterns.

At the landscape level, in addition to the aforementioned indices, SHDI and CONTAG were analysed to changes of overall landscape patterns from diversity and connectivity. SHDI characterizes the richness and evenness of landscape types, while CONTAG indicates the spatial aggregation and connectivity among patches. This multidimensional index system not only mitigates the limitations of single-dimensional metrics but also provides methodological support for a comprehensive understanding of spatiotemporal changes in landscape pattern, through the complementary nature of the selected indices.

The synchronous increase in PD and ED confirms the intensification of overall landscape fragmentation and shape complexity. The concurrent rise in SHDI (+ 5.3%) and decline in CONTAG (–3.25%) illustrate a problematic heterogeneous development trend in which landscape diversity increases through patch proliferation rather than deliberate diversification, while spatial connectivity is simultaneously impaired. These pattern changes are mechanistically linked to ecological security decline through four pathways: habitat isolation disrupting species dispersal, hydrological disruption reducing water availability, microclimate modification intensifying water stress, and cumulative ecosystem service loss. The stronger ecological security decline observed during 2000–2010 compared to other periods, coinciding with peak landscape fragmentation, is qualitatively consistent with theoretical predictions that fragmentation impacts are non-linear and disproportionately severe in arid systems with limited baseline connectivity, though quantitative regression between individual landscape metrics and the ecological security index remains a direction for future research to formally establish this mechanistic linkage.

The multidimensional landscape index constructed in this study not only effectively reveals the spatiotemporal evolution of landscape patterns in the study area but also provides a scientific basis for understanding the mechanisms through which land use change influences the ecological environment.

### Analysis of driving factors

Over the past 43 years, landscape pattern evolution in the Kashgar region has been influenced by both natural and human factors. These landscape pattern changes have subsequently exerted varying degrees of influence on ecological security^74^. Natural environmental factors, including climate, hydrology, soil, geology, and geomorphology, constitute the internal causes of regional landscape pattern change, with climate being the predominant element. In contrast, human factors such as population dynamics, economic structure, social development levels, and policy interventions represent external causes of landscape pattern changes and serve as the dominant driving forces.

#### Analysis of natural driving factors

The study area is situated in an arid region characterised by high sensitivity to climate change, where even minor climatic fluctuations can significantly affect the local ecological environment. It constitutes a typical mountain–plain–desert ecosystem, exhibiting distinct elevational zones and significant climatic gradients. Four representative meteorological stations were selected to capture regional climate variability: Kashgar (1288.7 m, in the north), Maralbishi (1116.5 m, in the east), Yarkant (1231 m, in the south), and Tashkorgan (3093.7 m, in the mountains)^53^.

**Fig. 10:**
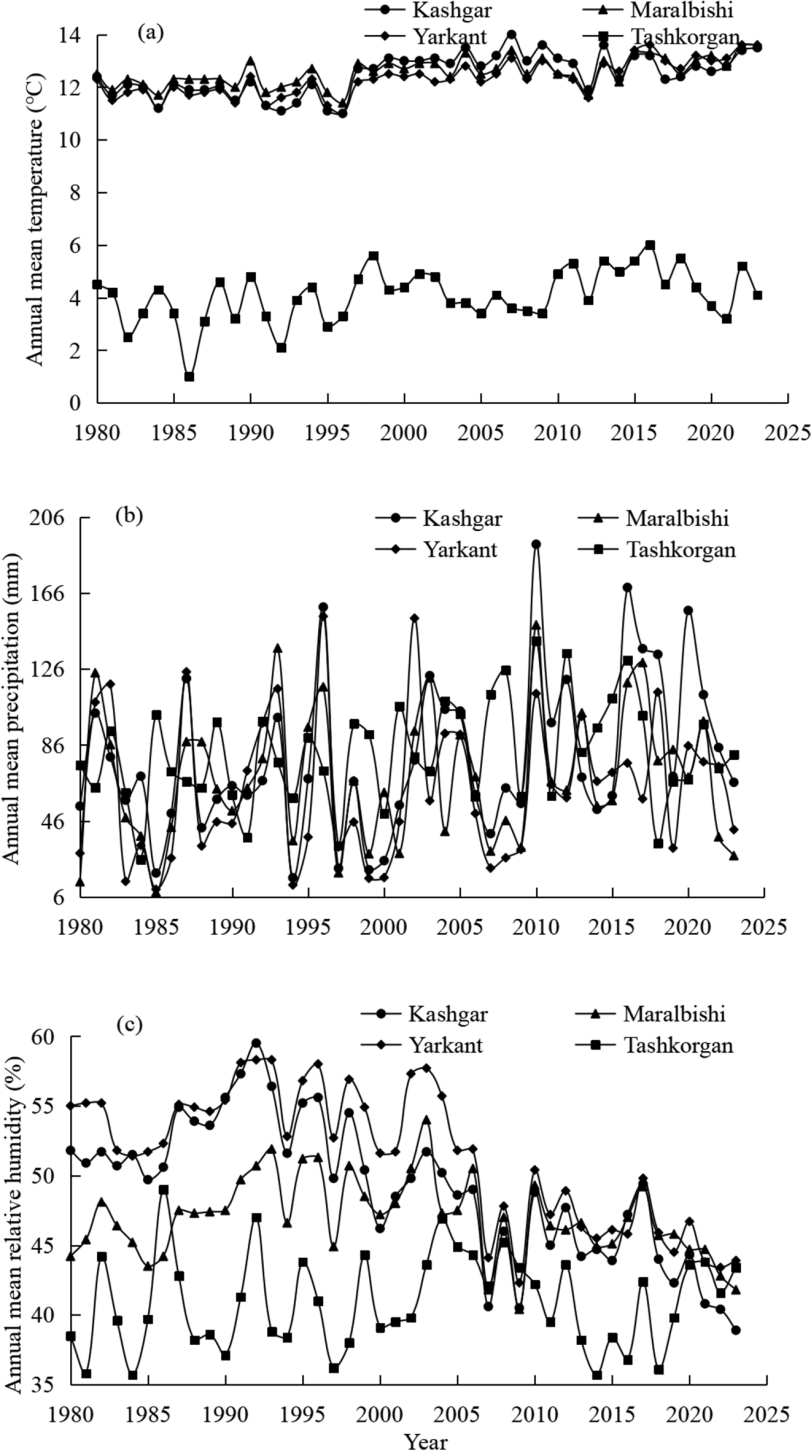
Temporal trends of natural factors (**a**) annual mean temperature, (**b**) precipitation, (**c**) relative humidity in the Kashgar region from 1980 to 2023.

Over the past 43 years, the Kashgar region (encompassing the four stations) has shown a clear warming trend in annual mean temperature (Fig. 10a), accompanied by a slight increase in annual mean precipitation (Fig. 10b). Annual mean relative humidity has generally decreased in the plains areas (Kashgar, Maralbishi, and Yarkant) but increased in the mountainous area (Tashkorgan) (Fig. 10c). In the plains, a slight increase in precipitation, rising temperatures and declining relative humidity resulted in enhanced evaporation from water bodies within the study area. This climatic change has induced a series of ecological responses: intensified surface water evaporation has led to marked shrinkage of water bodies such as lakes and reservoirs, along with diminished groundwater recharge. Concurrently, upward migration of soil salinity with evaporating moisture has expanded saline-alkali land areas. Furthermore, elevated temperatures have increased water demand for both vegetation and soil, exacerbating water deficit stress and resulting in vegetation degradation, reduced vegetation coverage, and decreased areas of forest and grass land.

To quantify these climate-land use relationships, Spearman correlation analysis was conducted between meteorological variables and land use change across the six study periods (n = 6, df = 4). Statistical significance was assessed using two-tailed tests with α = 0.05 (critical value t = 2.776). Annual mean temperature showed a significant negative correlation with water body area change (rs = –0.83, p < 0.05), confirming that warming was a primary driver of water body shrinkage. Relative humidity decline showed a positive association with unused land expansion (rs = 0.79, p = 0.06), though this relationship did not reach statistical significance at the α = 0.05 threshold; nonetheless, the observed trend is consistent with the interpretation that drying conditions may accelerate conversion of marginal lands. In contrast, the slight increase in precipitation showed no significant correlation with cultivated land expansion (rs = 0.41, p = 0.21), indicating that agricultural frontier expansion was driven primarily by human factors rather than improved water availability.

#### Analysis of human driving factors

**Fig. 11:**
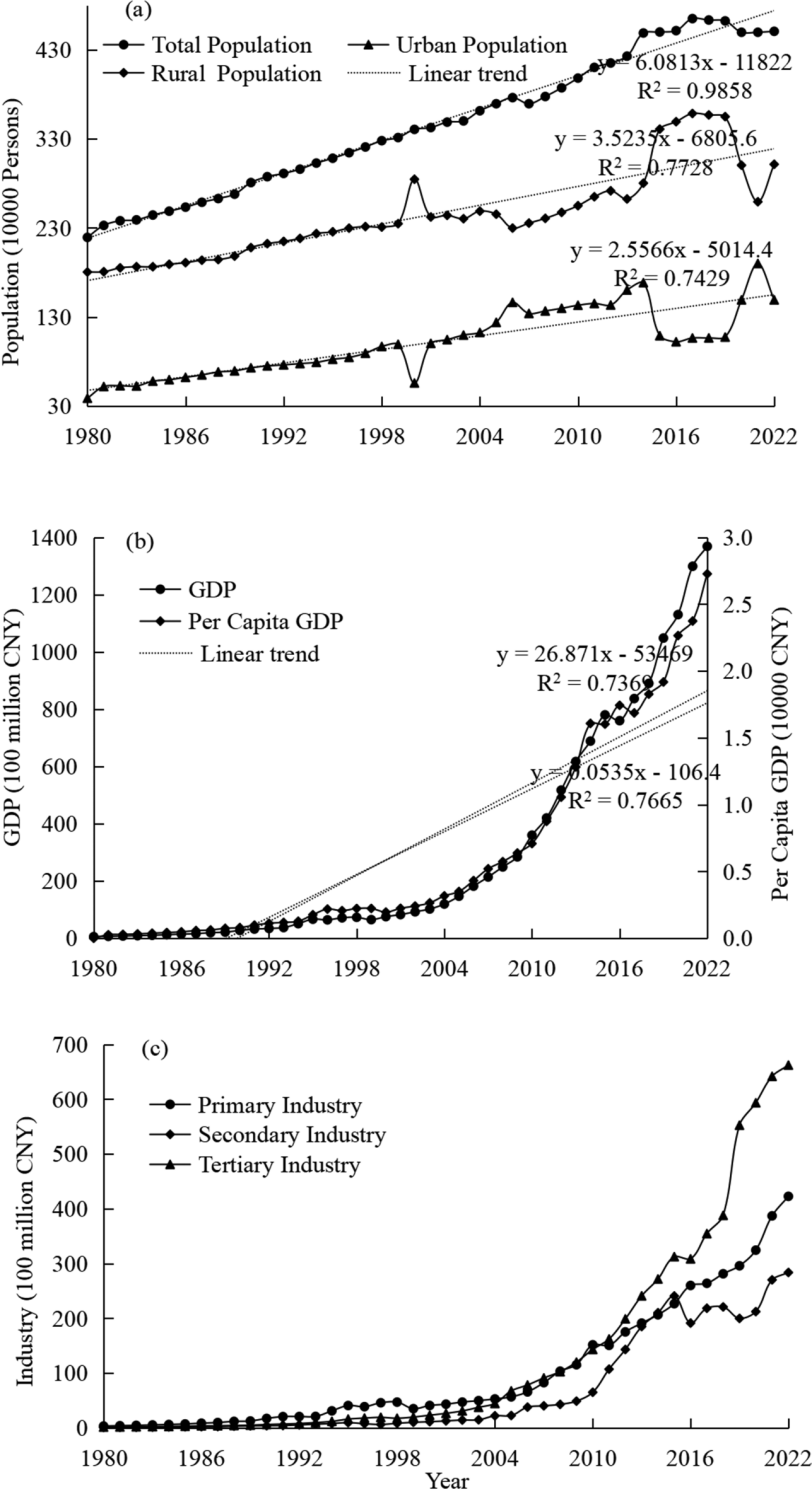
Temporal trends of human factors (**a**) population, (**b**) GDP & per capita GDP, (**c**) industry in the Kashgar region from 1980 to 2023.

From 1980 to 2022, the total population of the study area increased from 2.194 million to 4.507 million, representing a substantial growth of 2.313 million people over the 42-year period. The urban population grew by 47.72%, while the rural population increased by 52.27%. The regional gross domestic product (GDP) rose from 577 million CNY in 1980 to 136.856 billion CNY in 2022, with per capita GDP increasing from 263 CNY to 28,714 CNY (Fig. 11). Population growth and rapid economic development have sharply increased the demand for agricultural products, which existing cultivated land productivity could not fully meet. To fulfill basic survival and developmental needs, large-scale deforestation and reclamation of unused land were carried out to expand cultivated areas. Consequently, substantial areas of grass land, forest land, and unused land were converted to cultivated land within the study area. Meanwhile, growing demand for housing and industrial goods accelerated the expansion of urban, residential, and industrial-mining land. These changes demonstrate that population growth has directly driven the expansion of both cultivated and construction land.

To quantify the relative contributions of socioeconomic drivers, grey relational analysis (GRA) was performed between human factors (total population, GDP, per capita GDP) and land use changes across the study period. Results indicated that GDP growth exhibited the highest grey relational grade with construction land expansion (GRG = 0.87), followed by total population growth (GRG = 0.82), suggesting that economic development was the dominant anthropogenic driver of urbanization. For cultivated land expansion, total population showed the highest relational grade (GRG = 0.85), consistent with food security demands driving agricultural reclamation. Additionally, Spearman correlation confirmed significant positive relationships between total population and cultivated land area (rs = 0.94, p < 0.01), and between GDP and construction land area (rs = 0.96, p < 0.01). However, it must be acknowledged that GDP, total population, construction land, and cultivated land all exhibit monotonic upward trends across the 43-year period. Correlations of such magnitude may partially reflect shared temporal trajectories rather than exclusively causal driver-response relationships. While the manuscript addresses multicollinearity through Grey Relational Analysis, the specific issue of pseudocorrelation induced by common monotonic trends warrants explicit recognition. The exceptionally high correlation coefficients (rs > 0.94) should therefore be interpreted with caution, as they may overestimate the strength of direct causal linkages. It is acknowledged that GDP and total population are highly correlated in this dataset (rs = 0.96, p < 0.01), which limits the ability to fully isolate their independent contributions to land use change. Grey relational analysis was therefore applied as a relative ranking tool to compare driver importance rather than as a causal attribution method; multivariate regression controlling for collinearity would require a longer annual time series than the six decadal observations available in this study.

Regarding policy effects, the implementation of the Western Development Strategy in 2000 coincided with the most intense land use change period (2000–2010, *LC* = 1.20%), while the subsequent introduction of ecological red line policies after 2015 corresponded with decelerated land use change (2010–2020, *LC* = 0.15%), suggesting that governance interventions exerted measurable regulatory effects on landscape transformation trajectories.

Furthermore, policy interventions have been another key factor driving land use change in the Kashgar region, often resulting in significant adjustments to land use within relatively short timeframes. Since the implementation of the Western Development Strategy in 2000, rapid development has been effectively promoted in industry, infrastructure, urbanization, and ecological construction. In particular, large-scale implementation of policies such as the Grain for Green and water diversion projects have helped to slow the reduction of forest and grass land areas, while also contributing to the decline of unused land extent.

In summary, grey relational analysis and Spearman correlation collectively indicate that anthropogenic factors (GDP growth, population expansion, and policy interventions) exerted stronger and more direct influences on land use change than natural climatic factors, with GDP growth ranking as the single strongest driver of construction land expansion (GRG = 0.87) and population growth as the primary driver of cultivated land reclamation (GRG = 0.85). Natural factors, particularly temperature increase and relative humidity decline, acted as background modulators primarily affecting water body shrinkage and vegetation degradation rather than directly driving land conversion.

#### Coupled human-natural system dynamics and feedback mechanisms

The landscape-ecological security evolution in Kashgar exemplifies tele-coupling in border regions where local changes cascade across scales. Three feedback loops emerged:

Positive feedback (amplifying degradation): Water body shrinkage (–931.89 km^2^) → reduced regional humidity → accelerated evapotranspiration → further water loss. This vicious cycle is amplified by climate warming (+ 0.03°C/yr, Fig. 10a) and intensified by irrigation withdrawals for expanded cultivation (+ 3671.78 km^2^), creating a water-land-climate amplifier unique to arid oases.

Negative feedback (self-regulation): Policy interventions (the Grain for Green, 2000–) have provided significant impetus for ecological restoration, a notable recovery rate of 6.07% for forest land and 0.97% for grass land during 2020–2023. However, the total area of forest and grass land has not yet recovered to 2000 levels. This demonstrates that ecological restoration in arid regions is constrained not only by policy intensity, but more critically by the water carrying capacity in arid region constitutes an upper limit for self-regulation, resulting in a “restoration ceiling” effect.

Institutional feedback: A fourth mechanism emerges from our analysis. The observed inflection in forest recovery rates (6.07% during 2020–2023 versus –3.63% during 2000–2010) temporally coincides with China’s enhanced ecological compensation mechanisms and the establishment of ecological protection red lines under the 2018 territorial spatial planning reform. This suggests that governance innovations can modulate ecosystem trajectories, though the lag between policy implementation and ecological response (approximately 3–5 years based on our data) implies that adaptive management frameworks must anticipate rather than react to degradation signals.

Cross-scale interactions: County-level construction land expansion (+ 973.41 km^2^) exhibited spatially heterogeneous ecological impacts—concentrated in "high-high" clusters (Kashgar-Yarkant oases) it fragmented core habitats, while scattered growth in "low-low" zones (desert margins) paradoxically improved local patch diversity. This scale-dependent impact pattern challenges "one size fits all" policies.

### Critical threshold identification: Limitations and validation requirements

Throughout this manuscript, Δ*La* ≈ 2.5–3.5 per decade denotes the preliminary monitoring reference range, within which Δ*La* = 3.0 per decade serves as the central early-warning reference value for management purposes; Δ*La* > 3.0 per decade indicates the threshold above which immediate intervention is recommended. These formulations are complementary rather than contradictory and should be interpreted within the same conservative, pre-validation framework. The temporal dynamics during 2000–2010 reveal distinctive characteristics in land use change intensity. This period marked an exceptional inflection point, with Δ*La* reaching 3.31 and *R* reaching 2.14%—values that exceeded those in all other decades and coincided with China’s Western Development Strategy implementation. This intensity surge was accompanied by multiple ecological responses (fragmentation, connectivity loss, reduced restoration effectiveness) that are noteworthy for understanding landscape degradation dynamics.

However, identifying this period as a “threshold” requires careful qualification:

1. Sample limitation: The Δ*La* = 3.31 value is based on observation from a single study region (Kashgar) and a single time period (2000–2010). This is insufficient for establishing a general ecological threshold. Beyond the question of sample size, mechanistic interpretation also requires caution.
2. Mechanistic uncertainty: While the 2000–2010 period showed concurrent peaks in fragmentation (PD = 0.087), complexity (LSI = 73.95), and connectivity loss (CONTAG = 65.87%), we cannot definitively attribute these solely to Δ*La* = 3.31 without isolating its effects from confounding variables (climate, policy timing).
3. Recovery lag interpretation: The persistence of ecological degradation into 2010–2020 (despite declining land use change: *LC* = 0.15%) is consistent with hysteresis, but could also reflect slow continuous recovery rather than alternative stable states.

Therefore, we tentatively propose Δ*La* ≈ 3.0 per decade as a preliminary monitoring reference for early-warning systems in arid landscapes, subject to cross-regional validation, rather than a definitively identified ecological tipping point. Future research should:

- Conduct piecewise linear regression analysis across 5 + arid study regions.
- Test alternative stable state models using dynamical systems approaches.
- Compare degradation and recovery timescales to distinguish hysteresis from lag effects.
- Validate findings with controlled manipulation or quasi-experimental designs.

Based on this more conservative interpretation, the potential critical transition zone for arid landscapes is estimated at Δ*La* = 2.5–3.5 per decade, pending cross-regional validation.

This threshold hypothesis is suggested by three observational indicators drawn from the same single-region dataset, which cannot be treated as independent validation: (1) the 2000–2010 period with Δ*La* = 3.31 uniquely exhibited simultaneous increases in fragmentation (PD), complexity (LSI), and diversity (SHDI) while connectivity (CONTAG) declined—a pattern absent in other decades; (2) ecological security degradation persisted into 2010–2020 despite dramatically reduced land use change rates (*LC*: 1.20% → 0.15%), which is consistent with hysteresis but could alternatively reflect slow continuous recovery; (3) recovery timescales in the 2020–2023 period (partial) substantially exceeded disturbance timescales, qualitatively consistent with alternative stable state dynamics in semi-arid systems (Scheffer et al., 2009), though formal bistable modeling was not conducted. Cross-regional statistical validation using piecewise regression across multiple arid study areas is required before this observation can be elevated to a generalizable ecological threshold.

### Research limitations and prospects

This study has several limitations. (1) The land use data (30 m spatial resolution, 10-year intervals) have relatively coarse spatial and temporal resolution. Future studies could enhance the accuracy by employing higher-resolution imagery (e.g., Sentinel-2 at 10 m resolution) and applying continuous annual monitoring to better capture transient dynamics and abrupt transitions in landscape evolution. (2) Due to data availability constraints, not all relevant natural factors were incorporated. Consequently, the selection of driving factors may not be exhaustive. Influential elements such as annual runoff were not considered, despite their potential impact on landscape pattern evolution. (3) The selection of ecological security evaluation unit size varies across studies, with previous research employing units of 10 km^2^ × 10 km^2^ ^28,76^, 2 km^2^ × 2 km^2^ ^10^, 1 km^2^ × 1 km^2^ ^27^, and 200 m × 200 m ^74^. The 1 km^2^ unit adopted in this study was justified on methodological grounds (see Methods), and scale robustness was confirmed through comparison with 4 km^2^ and 9 km^2^ alternatives (Spearman rs > 0.93). Nevertheless, finer-resolution evaluation (e.g., 200 m × 200 m) combined with higher-resolution imagery may reveal sub-kilometer heterogeneity in ecological security patterns, representing a direction for future refinement.

Future research should use simulation prediction models (e.g., PLUS model) to investigate the spatial distribution of land use and the dynamic evolution of landscape ecological security under multiple scenarios for the year 2030. This endeavour would provide valuable insights for regional ecological governance and landscape optimisation, thereby promoting synergistic and sustainable development of ecological security and socio-economic systems.

Limitations specific to the proposed land use intensity monitoring threshold (Δ*La* ≈ 2.5–3.5 per decade) and directions for cross-regional validation have been discussed in detail in the preceding subsection ‘Critical threshold identification: Limitations and validation requirements‘ and are not repeated here.

## Conclusions

Based on land use data from 1980 to 2023 in the Kashgar region of Xinjiang, this study employed a multi-method approach encompassing analyses of land use dynamics, land use intensity index, transfer matrix, landscape pattern indices, landscape ecological security assessment, and spatial autocorrelation analysis. These methods systematically revealed the spatiotemporal evolution patterns of land use, landscape patterns, and landscape ecological security levels in the region. The conclusions are as follows: (1) The predominant land use types in the Kashgar region are unused land, grass land, and cultivated land, collectively accounting for 93.37% of the total study area. From 1980 to 2023, cultivated land and construction land expanded by 3671.78 km^2^ and 973.41 km^2^, respectively, while forest land, grass land, water bodies, and unused land decreased by 370.51 km^2^, 813.42 km^2^, 931.89 km^2^, and 2529.37 km^2^, respectively. (2) During the study period, significant variations were observed in the dynamics of different land use types. In terms of single land use dynamic degree, construction land exhibited the most pronounced changes, with a dynamic degree of 8.41%, followed by cultivated land and forest land. Water bodies, unused land, and grass land showed relatively lower dynamic degrees. The comprehensive land use dynamic degree exhibited a fluctuating trend from 1980 to 2023, peaking during 2000–2010 (LC = 1.20%) before declining markedly in 2010–2020 (LC = 0.15%). Land use intensity showed an overall increasing trend throughout the study period, with the most pronounced intensification occurring during 2000–2010, reflecting the progressively strengthening human impacts on the ecosystem. (3) From 1980 to 2023, construction land exhibited a persistent unidirectional inflow imbalance, while unused land showed a consistent unidirectional outflow imbalance. Cultivated land, forest land, grass land and water bodies engaged in bidirectional transitions with other land use types. (4) The Kashgar region exhibited increasing trends in patch density (PD), largest patch index (LPI), landscape shape index (LSI), edge density (ED), and shannon’s diversity index (SHDI) from 1980 to 2023, while the contagion index (CONTAG) declined. These changes indicate that the overall landscape pattern developed towards greater complexity, fragmentation, and dispersion. Among the various land use types, grass land and forest land exhibited the highest fragmentation, while the landscape shape and structure of water bodies and cultivated land became more complex. (5) Over the 43-year period, landscape ecological security displayed a fluctuating pattern of initial improvement, subsequent degradation, and eventual recovery. Although low and lower safety levels remained prevalent, the overall ecological security pattern showed a positive developmental trend. Spatial analysis revealed consistently positive *Moran’s I* values with intensified clustering effects, characterised by distinct "high-high" clusters centered in the Yarkant River and Kashgar oases, and "low-low" clusters in the ecologically fragile zone along the eastern fringe of the Taklimakan Desert. It should be noted that the proposed monitoring reference zone (Δ*La* ≈ 2.5–3.5 per decade) is derived from a single-region dataset with n = 6 observations, and should be interpreted as a preliminary hypothesis for early-warning management rather than a statistically validated ecological tipping point. Cross-regional validation using piecewise regression across multiple comparable arid systems is identified as a priority direction for future research.

## Supplementary Information

Supplementary Information.

## Data Availability

The datasets used and/or analyzed during the current study are available from the corresponding author on reasonable request.
