# Supplementary material for: Multi-decadal landscape dynamics and ecological security trajectories driven by 43-year land use changes in Kashgar, an arid border region of Northwest China
Source: Sci Rep. 2026 May 4;16:20495. doi: 10.1038/s41598-026-51246-y (PMC13328761; doi:10.1038/s41598-026-51246-y)
Supplement: Supplementary file 1 — Supplementary Information. [file 41598_2026_51246_MOESM1_ESM.docx]

**Table S1. Sensitivity analysis of landscape ecological security assessment to grid size and disturbance index weights**

| **Period** | **Grid Size** | **Weight Scheme** | **Moran's I** | **Rank Correlation with Baseline*** |
| --- | --- | --- | --- | --- |
| 1980 | 1 km² | Baseline (a=0.5, b=0.3, c=0.2) | 0.7842 | 1.000 |
|  | 1 km² | Scheme B (a=0.4, b=0.4, c=0.2) | 0.7856 | 0.9847 |
|  | 1 km² | Scheme C (a=0.6, b=0.2, c=0.2) | 0.7829 | 0.9823 |
|  | 4 km² | Baseline | 0.7798 | 0.9512 |
|  | 9 km² | Baseline | 0.7821 | 0.9387 |
| 1990 | 1 km² | Baseline | 0.7846 | 1.000 |
|  | 1 km² | Scheme B | 0.7861 | 0.9851 |
|  | 1 km² | Scheme C | 0.7834 | 0.9829 |
|  | 4 km² | Baseline | 0.7803 | 0.9523 |
|  | 9 km² | Baseline | 0.7825 | 0.9401 |
| 2000 | 1 km² | Baseline | 0.7850 | 1.000 |
|  | 1 km² | Scheme B | 0.7864 | 0.9856 |
|  | 1 km² | Scheme C | 0.7838 | 0.9831 |
|  | 4 km² | Baseline | 0.7809 | 0.9534 |
|  | 9 km² | Baseline | 0.7830 | 0.9415 |
| 2010 | 1 km² | Baseline | 0.8057 | 1.000 |
|  | 1 km² | Scheme B | 0.8069 | 0.9863 |
|  | 1 km² | Scheme C | 0.8045 | 0.9841 |
|  | 4 km² | Baseline | 0.8015 | 0.9547 |
|  | 9 km² | Baseline | 0.8038 | 0.9428 |
| 2020 | 1 km² | Baseline | 0.8001 | 1.000 |
|  | 1 km² | Scheme B | 0.8014 | 0.9859 |
|  | 1 km² | Scheme C | 0.7989 | 0.9837 |
|  | 4 km² | Baseline | 0.7961 | 0.9541 |
|  | 9 km² | Baseline | 0.7983 | 0.9422 |
| 2023 | 1 km² | Baseline | 0.8044 | 1.000 |
|  | 1 km² | Scheme B | 0.8057 | 0.9867 |
|  | 1 km² | Scheme C | 0.8032 | 0.9845 |
|  | 4 km² | Baseline | 0.8003 | 0.9551 |
|  | 9 km² | Baseline | 0.8026 | 0.9433 |

**Notes:**

*Rank correlation refers to Spearman's rs between ESk values computed under the alternative configuration and those from the baseline (1 km², a=0.5/b=0.3/c=0.2).

All Moran's I values were statistically significant (p < 0.001, Z > 2.58).

All rank correlations exceeded rs > 0.93 (p < 0.001), confirming robustness of conclusions to moderate variations in grid size and weight parameterization.

Weight schemes: a = fragmentation weight, b = separation weight, c = dominance weight (a+b+c=1).
